# Multiallelic, Targeted Mutagenesis of Magnesium Chelatase With CRISPR/Cas9 Provides a Rapidly Scorable Phenotype in Highly Polyploid Sugarcane

**DOI:** 10.3389/fgeed.2021.654996

**Published:** 2021-04-29

**Authors:** Ayman Eid, Chakravarthi Mohan, Sara Sanchez, Duoduo Wang, Fredy Altpeter

**Affiliations:** ^1^Agronomy Department, Institute of Food and Agricultural Sciences, University of Florida, Gainesville, FL, United States; ^2^Department of Energy Center for Advanced Bioenergy and Bioproducts Innovation, Gainesville, FL, United States; ^3^Genetics Institute, University of Florida, Gainesville, FL, United States; ^4^Plant Molecular and Cellular Biology Program, Institute of Food and Agricultural Sciences, Gainesville, FL, United States

**Keywords:** CRISPR/Cas9, genome editing, polyploid, magnesium chelatase, sugarcane, biolistic gene transfer, heat treatment

## Abstract

Genome editing with sequence-specific nucleases, such as clustered regularly interspaced short palindromic repeats (CRISPR)/CRISPR-associated protein 9 (Cas9), is revolutionizing crop improvement. Developing efficient genome-editing protocols for highly polyploid crops, including sugarcane (*x* = 10–13), remains challenging due to the high level of genetic redundancy in these plants. Here, we report the efficient multiallelic editing of magnesium chelatase subunit I (*MgCh*) in sugarcane. Magnesium chelatase is a key enzyme for chlorophyll biosynthesis. CRISPR/Cas9-mediated targeted co-mutagenesis of 49 copies/alleles of magnesium chelatase was confirmed via Sanger sequencing of cloned PCR amplicons. This resulted in severely reduced chlorophyll contents, which was scorable at the time of plant regeneration in the tissue culture. Heat treatment following the delivery of genome editing reagents elevated the editing frequency 2-fold and drastically promoted co-editing of multiple alleles, which proved necessary to create a phenotype that was visibly distinguishable from the wild type. Despite their yellow leaf color, the edited plants were established well in the soil and did not show noticeable growth retardation. This approach will facilitate the establishment of genome editing protocols for recalcitrant crops and support further optimization, including the evaluation of alternative RNA-guided nucleases to overcome the limitations of the protospacer adjacent motif (PAM) site or to develop novel delivery strategies for genome editing reagents.

## Introduction

The processing of sugarcane (*Saccharum* spp. hybrid) biomass provides 80% of the sugar and 26% of the ethanol produced globally. Sugarcane is one of the most productive crops under cultivation due to its superior light conversion and efficiencies of water and nitrogen use (Tew and Cobill, 2008; Byrt et al., 2011). It is also a prime candidate feedstock for the emerging bio-economy (Altpeter and Ratna, 2018). The highly polyploid (x = 10 − 13; 2n = 100 − 130), heterozygous, interspecific, and aneuploid sugarcane genome decelerates attempts at crop improvement (Le Cunff et al., 2008; de Setta et al., 2014). Most parental sugarcane clones lack pollen fertility and any synchrony of flowering, posing challenges to conventional breeding (Moore and Nuss, 1987; Horsley and Zhou, 2013). Elite cultivars display a high level of heterozygosity and polyploidy, requiring vegetative propagation to prevent the loss of favorable alleles and the accumulation of detrimental ones during the disruptive process of meiosis. Therefore, adding superior alleles to improve an elite cultivar with the use of conventional breeding is a demanding and time-consuming undertaking. Genome editing using sequence-specific nucleases (SSNs) is a powerful approach for the genetic improvement of crops (Zhang et al., 2018). It has great potential for sugarcane and other vegetatively propagated, heterozygous, and polyploid crops (Weeks, 2017) by enabling precision genome modifications in elite varieties while bypassing meiosis. Among the SSNs, RNA-guided nucleases, including CRISPR/Cas9, are the most widely used gene editing tools due to their target specificity, efficiency, simplicity of design, multiplexing capacity, and versatility (Chandrasegaran and Carroll, 2016). They have been repurposed to targeted mutagenesis, gene stacking, targeted nucleotide substitutions, chromosomal translocations, transcriptional or translational regulation, and viral interference (Jinek et al., 2012; Shan et al., 2013; Ali et al., 2015; Baltes et al., 2015; Svitashev et al., 2015; Zhang et al., 2018; Huang and Puchta, 2019; Beying et al., 2020; Gao et al., 2020).

Most approaches to genome editing require a DNA double-strand break (DSB) in or near the target sequence to be edited. *Streptococcus pyogenes* Cas9 (spCas9) possesses innate nuclease activity, which is targeted by an engineered, single 20 nt guide RNA molecule to the DNA cleavage site adjacent to a protospacer-associated motif (PAM) (Jinek et al., 2012). Then DNA cleavage triggers cellular repair mechanisms, including non-homologous end joining (NHEJ), microhomology-mediated end joining (MMEJ), and homology-directed repair (HDR), to rectify the DSB. The error-prone NHEJ and MMEJ repair pathways enable the construction of knockout alleles through frameshift mutations caused by indels. By contrast, HDR supports precision edits, including targeted codon replacements and gene stacking. HDR relies on recombination, using a template that displays homology to the break site (Puchta, 1998; Shrivastav et al., 2008; Chapman et al., 2012; Butt et al., 2019; Huang and Puchta, 2019).

Targeted mutagenesis is more challenging in highly polyploid crops such as sugarcane than in diploid crops. The large number of homeologs and homologs in sugarcane causes functional redundancy. However, this also offers an opportunity to generate a range of phenotypes, depending on the number of co-mutated copies/alleles, similar to RNAi. The creation of knockdown or knockout phenotypes requires an efficient multiallelic editing platform. We recently reported the TALEN-mediated targeted co-mutagenesis of more than 100 copies/alleles of the lignin biosynthetic gene *caffeic acid O-methyltransferase* (*COMT*) in sugarcane. This action resulted in drastically improved saccharification efficiency and greater bioethanol yields from the lignocellulosic biomass without compromising agronomic performance (Jung and Altpeter, 2016; Kannan et al., 2018; Ko et al., 2018).

The establishment of CRISPR/Cas9-mediated genome editing for sugarcane is desirable for improving multiplexing capacity, versatility, and ease of design relative to TALEN (Eid and Mahfouz, 2016). This will involve the optimization of genome editing reagents and their delivery to enable efficient co-editing of a large number of copies/alleles.

**Figure 1:**
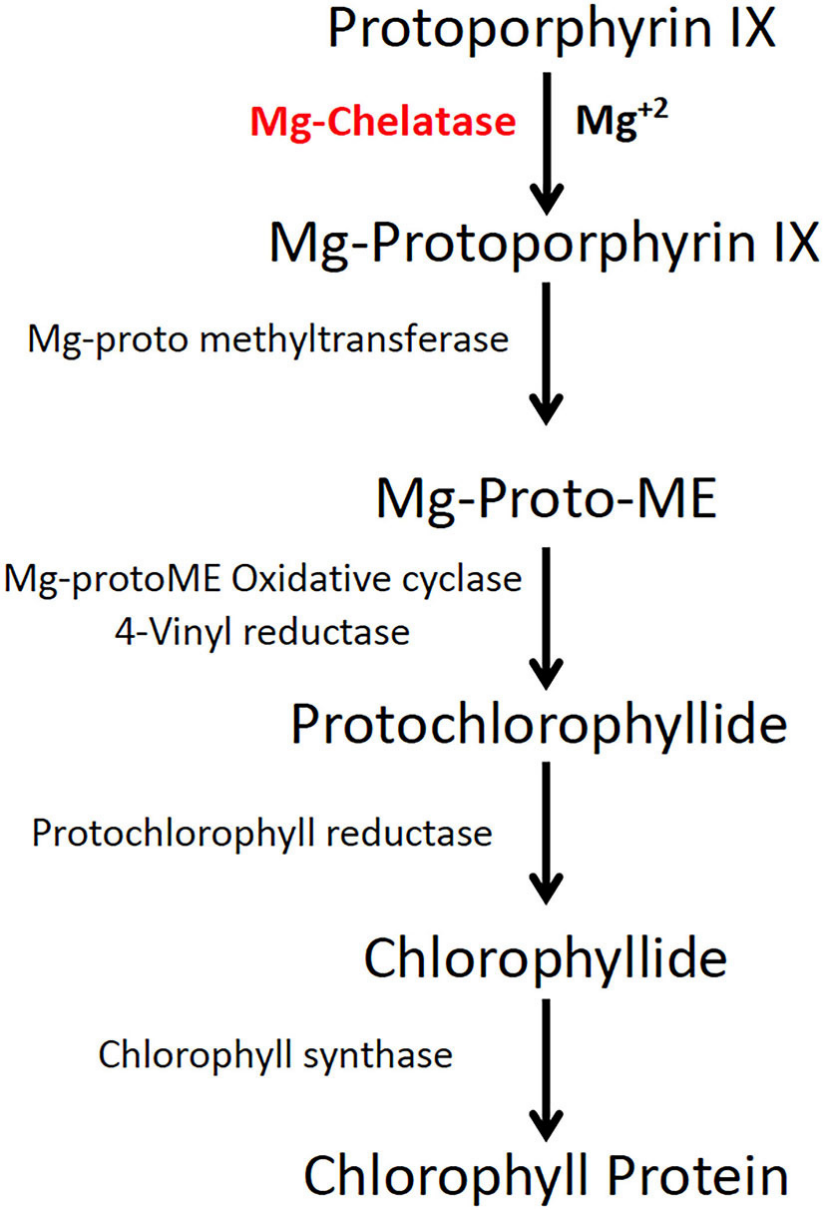
Magnesium chelatase is a key enzyme in the chlorophyll biosynthesis pathway. Magnesium chelatase catalyzes the conversion of protoporphyrin IX (PPIX) to magnesium protoporphyrin IX, a precursor of chlorophyll b and a in the presence of Mg^2+^ and ATP. The enzyme is a hexameric motor complex made up of three proteins, ChlI, ChlD, and ChlH.

These optimizations are accelerated with the help of a rapidly scorable screening system that allows the visual identification and quantification of targeted mutations as soon as plants regenerate from tissue cultures. To establish genome editing protocols in other crops, the phytoene desaturase (*PDS*) gene in the carotenoid biosynthetic pathway was targeted for mutagenesis (Shan et al., 2013; Fan et al., 2015). Unlike dwarf and albino phenotypes of PDS knockouts, Mg-chelatase mutants display a light green to yellow leaf phenotype with similar growth rates to the wild type (WT) (Walker et al., 2018). Mg-chelatase catalyzes Mg^2+^ attachment to protoporphyrin IX, which is the major regulatory point for the chlorophyll biosynthesis pathway (Figure 1) (Willows et al., 1996).

In this study, we explored whether targeted mutagenesis of magnesium chelatase subunit I with CRISPR/Cas9 provides a rapidly scorable phenotype for predicting the extent of multiallelic editing in highly polyploid sugarcane.

## Materials and Methods

### Isolation of Allelic Variants of Mg Chelatase and Design of sgRNAs

**Figure 2:**
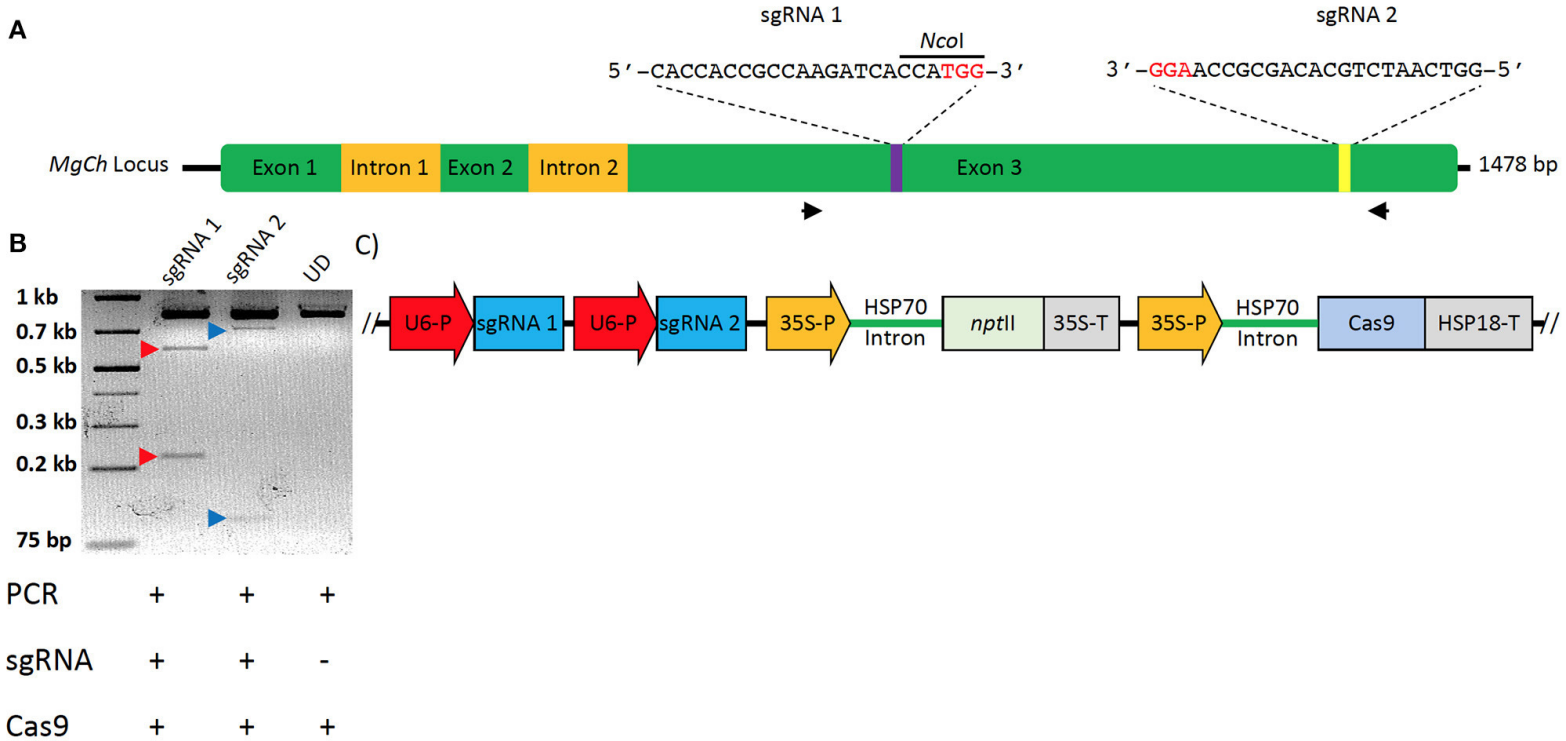
Strategy for targeted mutagenesis of sugarcane *MgCh* and confirmation of *In-vitro* cleavage activity of sgRNAs. **(A)** Schematic representation of sugarcane *MgCh* locus and sgRNAs' target sites, sgRNA1 targeting nts 731–750 and sgRNA2 targeting nts 1223–1242. Mutations at sgRNA target site 1 would disrupt the *Nco*I restriction recognition site. Exons are indicated with green boxes, and introns with yellow boxes. **(B)** *In vitro* cleavage assay to validate sgRNA activity, the (810 nts) *MgCh* PCR amplicon is digested by ribonucleoprotein complex (RNP) of Cas9 and either sgRNA1 or sgRNA2 into ~581 and 229 nts or 701 and 109 nts, respectively. **(C)** Map of sugarcane gene editing plasmid (pMGE); Two sgRNAs are monoscistronically expressed under *Oryza sativa* U6 promoter, *npt*II is under transcriptional control of cauliflower mosaic virus (CaMV) 35S promoter and CaMV terminator, Cas9 is under transcriptional control of CaMV 35S promoter and *Sorghum bicolor* HSP18 terminator. Protospacer adjacent motifs (PAMs) are indicated in red font.

The *MgCh* sequence in sugarcane was compared to sorghum and maize *MgCh* sequences via tBLASTn. This allowed the conserved domains to be identified, informing the primer design for the PCR amplification of multiple allelic *MgCh* variants from sugarcane target cultivar CP88-1762 (WT) (Supplementary Table 1). The amplicons were cloned into the p-GEMT® easy vector (A1360) (Promega, WI, USA), followed by the Sanger sequencing of multiple colonies. The sgRNAs were selected *in silico* using CRISPOR (http://crispor.tefor.net/). sgRNA1 was designed to cleave to a highly conserved region, while the sgRNA2 target was less conserved and included three allelic variants that differed in the number of mismatches (0, 1, or 2) in the genomic target sequence of the sgRNA (Figure 2A).

### sgRNA Synthesis and *in vitro* Cleavage Assay

sgRNA templates were generated via PCR using Q5® High-Fidelity DNA Polymerase (NEB, MA, USA) using a DNA template encoding T7 promoter sequence corresponding to the target sequence. The optimized sgRNA scaffold (Chen et al., 2013) was assembled from oligonucleotides (Eurofins Genomics, KY, USA) through overlapping PCR, as previously described (Lin et al., 2014). Primer T7MgCh1F was combined with T7F, ScaffoldR1, and ScaffoldR2 to generate sgRNA1 DNA. The primer T7MgCh2F was combined with T7F, ScaffoldR1, and ScaffoldR2 to generate sgRNA2 DNA using the following PCR conditions: 30 cycles of 95°C for 10 s, 57°C for 10 s, and 72°C for 10 s (Lin et al., 2014). The reactions were purified with the GeneJET PCR Purification Kit (K0701) (Thermo Fisher Scientific, MA, USA) and were electrophoresed with 1% agarose gel. *In vitro* transcription was done using the HiScribe™ T7 Quick High Yield RNA Synthesis Kit (E2050S) (NEB) with 75 ng sgRNA DNA template. DNase I treatment and RNA cleanup were performed using Monarch® Total RNA Miniprep Kit (T2010) (NEB). The Mg-chelatase template PCR was amplified using Q5® High-Fidelity DNA Polymerase (M0491) (NEB) with primers C27 and C28 and the following cycle: initial denaturation at 98°C for 30 s, then 35 cycles of 98°C for 5 s, 68°C for 10 s, and 72°C for 20 s, with a final extension of 72°C for 2 min. sgRNAs validation was done by incubating 200 ng Mg-Chelatase template DNA, 250 ng sgRNA, 250 ng Cas9 protein (PNA Bio), and 2 μL NEB buffer 3 in a 20 μL reaction for 3 h at 37° C. The reaction was stopped with the addition of 1 μL PureLink™ RNase A (20 mg/mL) (12091021) (Thermo Fisher Scientific) and was incubated for 10 min at 65°C prior to electrophoresis with 2% agarose gel (Figure 2B).

### Vector Construction

sgRNA vectors containing *Oryza sativa* U6 promoter were designed and custom synthesized in the pUC57 backbone (Genscript, NJ, USA) to generate pUCMg12. The Mg-chelatase target guide sequences were simultaneously cloned into the vector using annealed primer-dimers (Supplementary Table 1) holding 5′ overhangs to ligate into *Bbs*I and *Bsa*I restriction sites of pUC57. pUCMg12 vector was subcloned into the CRISPR backbone vector through digestion with *Srf* I and *Not*I enzymes, and the resultant colonies were confirmed via Sanger sequencing. The resulting vector MGE harbors a sugarcane codon-optimized *Streptococcus pyogenes* Cas9 driven by CaMV35S promoter with ZmHSP70 (heat shock protein) intron and AtHSP terminator. pMGE expresses the *npt*II (neomycin phosphotransferase II) gene as a plant selectable marker under transcriptional control of CaMV35S promoter with ZmHSP70 intron and CaMV35S terminator (Figure 2C).

### Biolistic Transformation of Sugarcane

The MGE plasmid was linearized using the *Asc*I enzyme, and the minimal cassette was introduced into the embryogenic callus of sugarcane cultivar CP88-1762 through biolistic gene transfer, as described previously (Taparia et al., 2012). To evaluate the impact of heat treatment on mutation frequency, 50% of the calli were heat-treated at 37°C for 48 h, 4 days after bombardment, and they were compared to bombarded calli from the same experiment that were kept at the usual incubation temperature (28°C). The calli were subsequently incubated at 28°C and selected with geneticin (20 mg/L), as described by Taparia et al. (2012). Plantlets 5–10 cm in height were sampled for molecular analyses.

### DNA Isolation, PCR, and Sanger and Next-Generation Sequencing

Genomic DNA was isolated from leaf tissues using the CTAB method (Murray and Thompson, 1980). The C5 and C9 primers (Supplementary Table 1) used for PCR amplified a region of *MgCh*, spanning exon 3 (~1,100 bp) from the genomic DNA template, including targets for sgRNAs 1 and 2 (Figure 2A). Q5® High-Fidelity DNA Polymerase (NEB, MA, USA) was used for PCR under the following conditions: 98°C for 30 s, 35 cycles of amplification at 98°C for 15 s, 55°C for 10 s, 72°C for 20 s, and final extension at 72°C for 2 min. The PCR amplicons used in the restriction enzyme assays to detect targeted mutations were amplified using C27 and C28 primers (Supplementary Table 1) and the Phire Plant Direct PCR Kit, under the following conditions: 98°C for 5 min, 35 cycles of amplification at 98°C for 5 s, 65.1°C for 5 s, 72°C for 20 s, and final extension at 72°C for 2 min, using PCR amplicons for Sanger sequencing ligated to pJET 1.2 blunt vector (Thermo Fisher Scientific, MA, USA). The plasmid DNA was prepared from cloned amplicons using the GeneJET miniprep kit (Thermo Fisher Scientific). Sanger sequencing of the cloned PCR amplicons was performed at Eurofins Genomics. The sequence chromatograms were visually checked for quality.

For next-generation sequencing, amplicons of 574 bp were generated using primers C31 and C32 (Supplementary Table 1) with Phire polymerase (Thermo Fisher Scientific) and the following amplification conditions: 98°C for 5 min, 35 cycles of amplification at 98°C for 5 s, 64.2°C for 5 s, 72°C for 20 s, and a final extension at 72°C for 2 min. The reactions were purified using a GeneJET PCR Purification Kit (K0701) (Thermo Fisher Scientific). Complete amplicon sequencing was performed using the CCIB DNA Core Facility at Massachusetts General Hospital (Cambridge, MA). Adapters with unique barcodes were ligated onto each sample during the library construction. The libraries were pooled into equimolar concentrations for multiplexed sequencing on the Illumina MiSeq platform (Illumina, San Diego, CA) with 2×150 run parameters.

To detect edited sgRNA target sites, all reads were examined in the corresponding fastq file to identify either the 5′ primer (C31) or the 3′ primer (C32) close to the beginning or the end of the read, respectively (exact match required). If primer C31 was found, a local alignment algorithm was run with the parameters match score = 1, mismatch penalty = −0.5, gap opening penalty = −0.5, and gap extension penalty = −0.2 to search for the sgRNA1 sequence in the 65 bp region downstream of C31. A minimum score of 15 was required to accept the alignment, in addition to a perfect match on the first two bases. If C32 was found, a search for the sgRNA2 sequence in the 50 bp region downstream of C32 was initiated, with the same parameters. If a match for the sgRNA sequence was found, the alignment was examined to determine the number of base substitutions, insertions, and deletions, and the number of reads that contained every possible combination of events (e.g., perfect match, substitutions only, substitutions and insertions, substitutions and deletions, etc.) was reported. In addition, matrices showing the frequencies of all observed substitutions in each sample were generated.

For the sgRNA2 target, the results were computed separately for each of the three known sgRNA2 variants, based on single-nucleotide changes at positions 4 (C → T) and 15 (T → C). Only the three haplotypes CT, TT, and TC were observed at a significant frequency. To determine the indel sizes, the number of reads in which the sgRNA target sequence contained insertions (or deletions) totaling 1, 2, 3, 4, 5, or more than 5 base pairs (based on the local alignment) were also reported, as well as the average size of all insertions or deletions. The results for each of the three sgRNA2 variants were reported separately. To record the reads with severely modified sgRNA targets while preventing alignment with the sgRNA sequence, all reads that contained a valid primer (C31 or C32) but did not contain the sgRNA sequence were examined. This included searching for an 11 bp conserved sequence that could be located downstream of the sgRNA (at positions 56 and 61 downstream of C31 or C32, respectively). This search was performed using a local alignment algorithm with the following parameters: match score = 1, mismatch penalty = −1, gap opening penalty = −2, gap extension penalty = −2, and minimum score required for hit = 9. All analyses were performed with custom Python scripts using the Biopython package (specifically, the Bio.pairwise2 library).

### Detecting Targeted Mutations Using Restriction Enzyme Digestion of PCR Amplicons

PCR products from C27–C28 were purified using GeneJET PCR Purification Kit (K0701) (Thermo Fisher Scientific). Following this, 200 ng of each purified product was incubated with 0.2 μL *Nco*I-HF (NEB) for 3 h at 37°C. The reactions were deactivated by incubation at 80°C for 20 min prior to loading on 2% agarose gel for visualization.

### Phenotypic Evaluations

The plants were transferred from tissue culture media to Sunshine mix #8 (Sungrow Horticulture, Agawam, MA, USA) potting mix and grown in a walk-in growth chamber when the shoots were ~10 cm long. During the first week after the transfer, a level of relative humidity near 100% was maintained, and then it was adjusted to 75% humidity. Plant growth occurred on a 16/8 h light/dark photoperiod and a light intensity of 400 μmol m^−2^ s^−1^, at 28/22°C day/night temperature. The plants were fertilized every 2 weeks after their transfer to the soil by irrigation with Miracle Grow All Purpose Plant Food (ScottsMiracle-Gro, Maryville, OH, USA). Leaf greenness was measured on the fully expanded top leaf from three tillers per plant using a SPAD chlorophyll meter (Minolta SPAD-502, Konica-Minolta), and this was repeated twice at 3-week intervals.

### Statistical Analyses

The means were compared using Fisher's least significant difference test. A minimum of three independent biological replicates were used for the statistical analyses.

## Results

### Identification of Allelic Variants of *MgCh* in Sugarcane and Confirming sgRNA Activity *in vitro*

Sanger sequencing of cloned *MgCh* amplicons from WT DNA led to the identification of allelic variants in WT and informed the design of two sgRNAs that targeted exon 3. sgRNA1 targeted *MgCh* at nts 731–750, and sgRNA2 targeted *MgCh* at nts 1223–1242 (Figure 2A). Both sgRNAs were validated through *in vitro* cleavage assay. sgRNA1 guided the cleavage of the 810 nt long partial *MgCh* amplicons into 581 and 229 nt fragments. Targeting via sgRNA2 generates 701 and 109 nts fragments upon cleavage (Figure 2B).

### Visual Detection of Mutant Phenotypes

**Figure 3:**
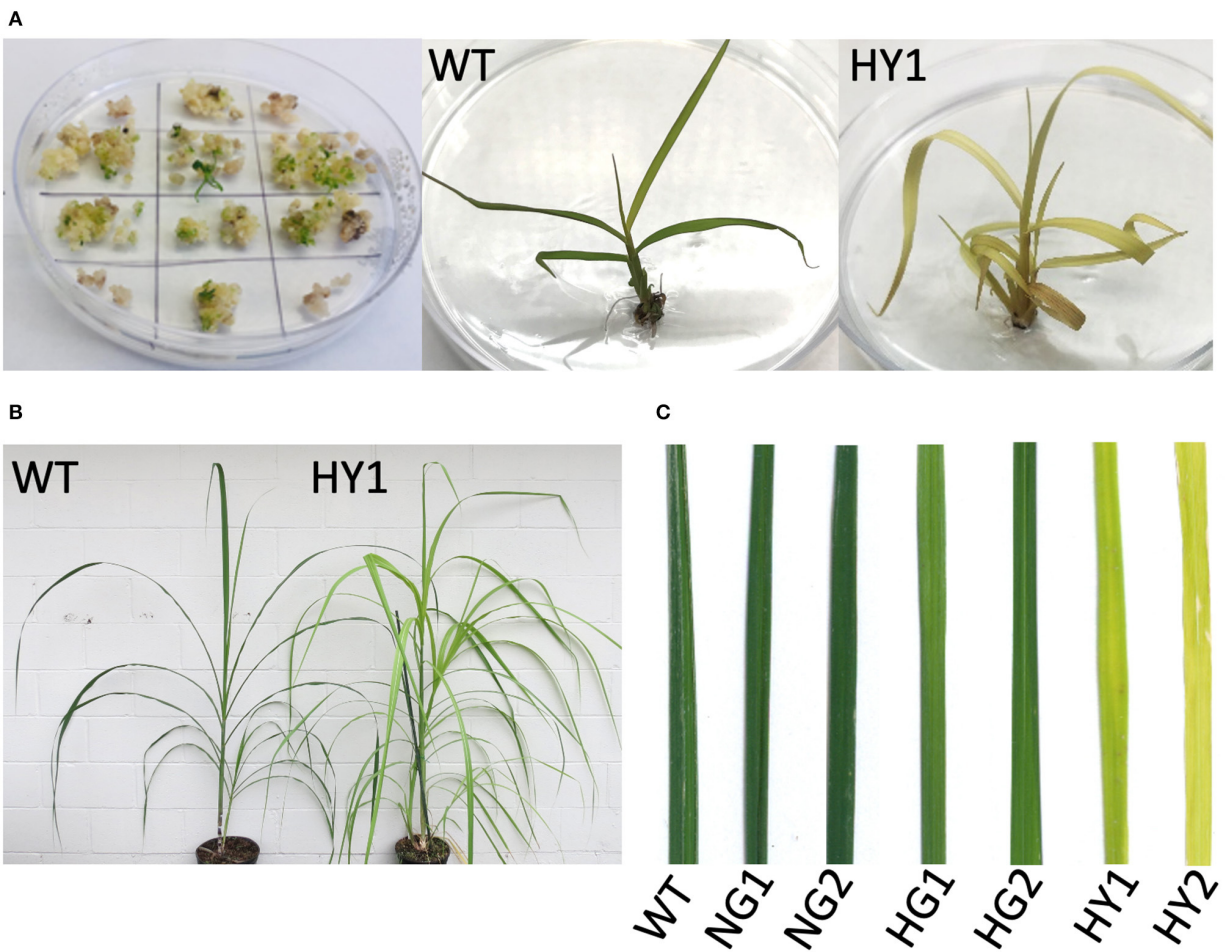
Detection of chlorophyll depletion phenotype during *in vitro* propagation. **(A)** Development of chlorophyll depletion phenotype compared to the WT. Some of the genome-edited events (e.g., line HY1 [heat-treated, yellow 1]) displayed a distinguishable phenotype with yellow leaf color in plantlets that regenerated from tissue culture. **(B)** Comparison of line HY1 (right) to WT (left) after establishment in soil under greenhouse conditions. **(C)** Close-up of leaves from NG1, NG2, HG1, HG2, HY1, and HY2 compared to WT. HY = heat-treated yellow, NG = non-heat treated green, HG = heat treated green, WT = wild type.

Events that feature the depletion of chlorophyll are visually distinguishable as soon as the plantlets regenerate from tissue culture (Figure 3A). This included light green or yellow leaves, by contrast to the dark green shoots from the non-bombarded control plates.

### Characterization of Mutant Lines With Restriction Enzyme Assay

**Figure 4:**
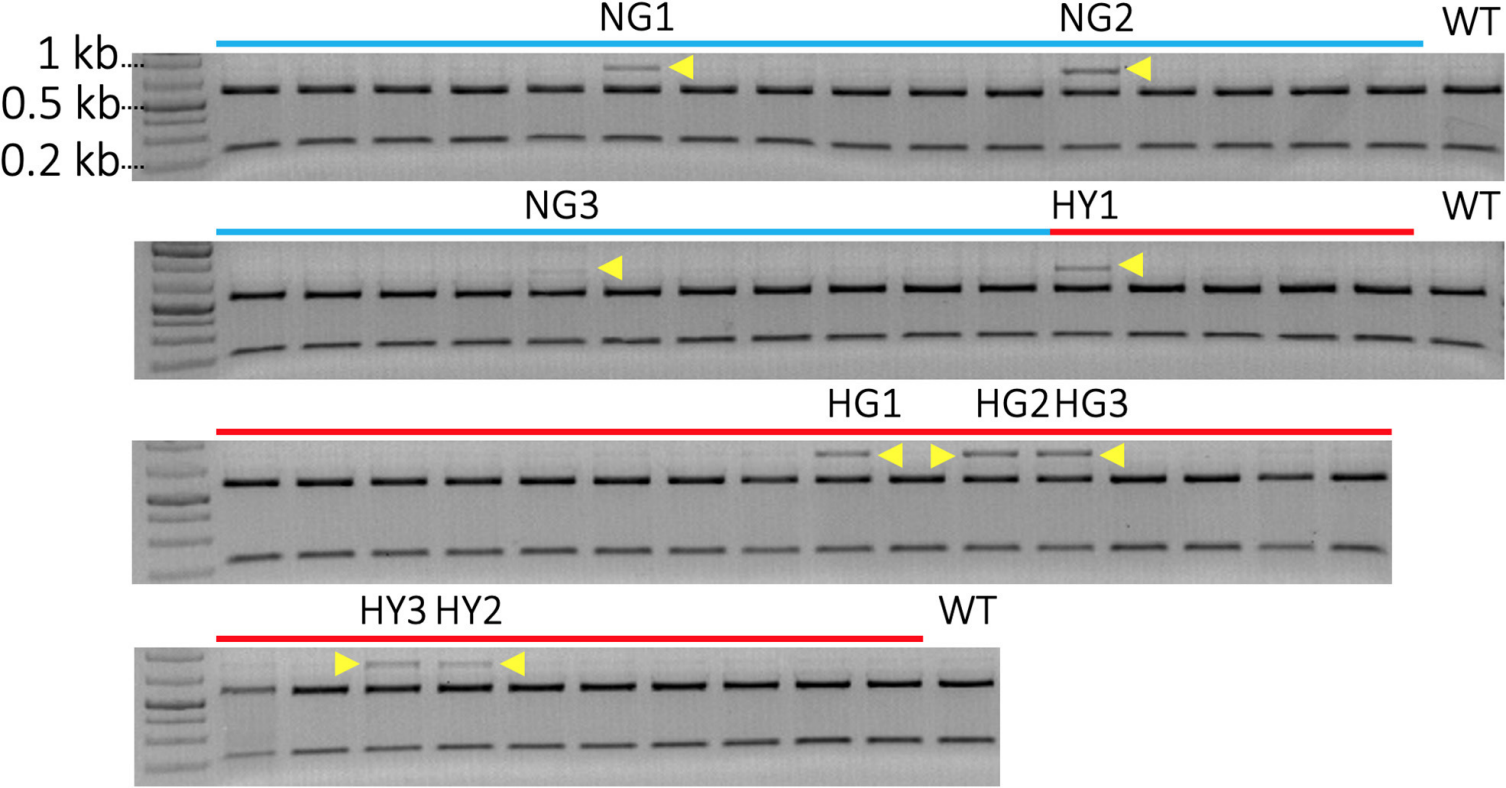
Identification of mutants by detection of restriction site loss in sgRNA1 target site of *MgCh* PCR amplicons. Cleavage in sgRNA1 target site is likely to disrupt *Nco*I restriction site due to the creation of indels through an error-prone NHEJ-mediated repair process. A functional *Nco*I site would allow the cleavage of 810 bp PCR amplicon of *MgCh* into ~578 bp and 232 bp products. Blue lines indicate transgenic lines generated without heat treatment, and red lines indicate transgenic lines that regenerate following heat treatment of bombarded callus at 37°C for 48 h. N = non-heat treated, G = green, H = heat-treated, Y = yellow. The *Nco*I digested *MgCh* PCR amplicon was electrophoresed on 2% agarose gel. Lines NG1, NG2, NG3, HY1, HG1 HG2, HG3, HY3, and HY2 all display partially undigested *MgCh* PCR amplicons following *Nco*I restriction digestions indicated by yellow arrows. HY = heat-treated, yellow; NG = non-heat treated, green; HG = heat treated, green; WT = wild type.

A total of 52 transgenic lines were regenerated, including 22 lines from non-heat-treated tissue and 30 lines from heat-treated tissue from 10 bombardments with the pMGE construct for each treatment. The PCR amplicons of *MgCh* from these lines were analyzed for the loss of restriction sites in the target region of sgRNA1. Loss of the *Nco*I restriction site was expected if indels or nucleotide substitutions were generated in the target site of the sgRNA1 (Figure 2A). A total of nine lines were identified, including a partially undigested *MgCh* amplicon following *Nco*I treatment, by contrast to WT, which displayed a completely digested amplicon (Figure 4). Among the nine lines with altered *Nco*I restriction digest pattern, three lines originated from no-heat treatment (NG1, NG2, and NG3), and six lines were from heat-treated calli (HY1, HY2, and HY3 and HG1, HG2, and HG4; Figure 4).

### Sanger Sequencing of Cloned PCR Amplicons of *MgCh*

Sanger sequencing of cloned PCR amplicons, including the 2 sgRNA target regions in exon 3 of *MgCh* (Figure 2A), confirmed the targeted mutations from the non-heat-treated set. Three mutant lines were confirmed by Sanger sequencing and restriction enzyme assay (14% of the regenerated lines; Table 1 and Supplementary Figure 1). The heat-treated tissues regenerated six mutant lines (20% of the regenerated lines, Table 1 and Supplementary Figure 1). All three of the lines with the most severe chlorophyll depletion HY1, HY2, and HY3 (heat-treated yellow 1, 2, and 3; Table 1) were derived from the heat treatment. In addition, three mutants with mild to moderate chlorophyll reduction were also regenerated from the heat treatment (HG1, HG2, and HG3; Table 1).

**Table 1:** Summary of phenotyping and genotyping of regenerated plants following biolistic transfer of pMGE and treatment with 37°C or regular incubation temperature (28°C).

**Temperature treatment**	**No. of shots**	**Cas9 (+ve) lines**	**Yellow Leaves**	**Mosaic Leaves**	**RSL (+ve)**	**Sanger (+ve)**	**Editing frequency/Shot**	**Editing frequency/Cas9 lines**
28°C	10	22	0	0	3	3	3/10 (30%)	3/22 (14%)
37°C	10	30	3	1	6	6	6/10 (60%)	6/30 (20%)

*Number of regenerated, transgenic, and edited lines following the biolistic transfer of pMGE minimal cassette and 48 h treatment with 37°C or regular incubation temperature (28°C). (+v) = PCR positive, RSL = restriction site loss*.

Single-nucleotide polymorphism outside of the target regions for sgRNA1 and sgRNA2 allowed the identification of unique reads to represent individual *MgCh* copies/alleles. This allowed differentiation between single-edited and co-edited events within *MgCh* variants/alleles in the analyzed lines.

**Figure 5:**
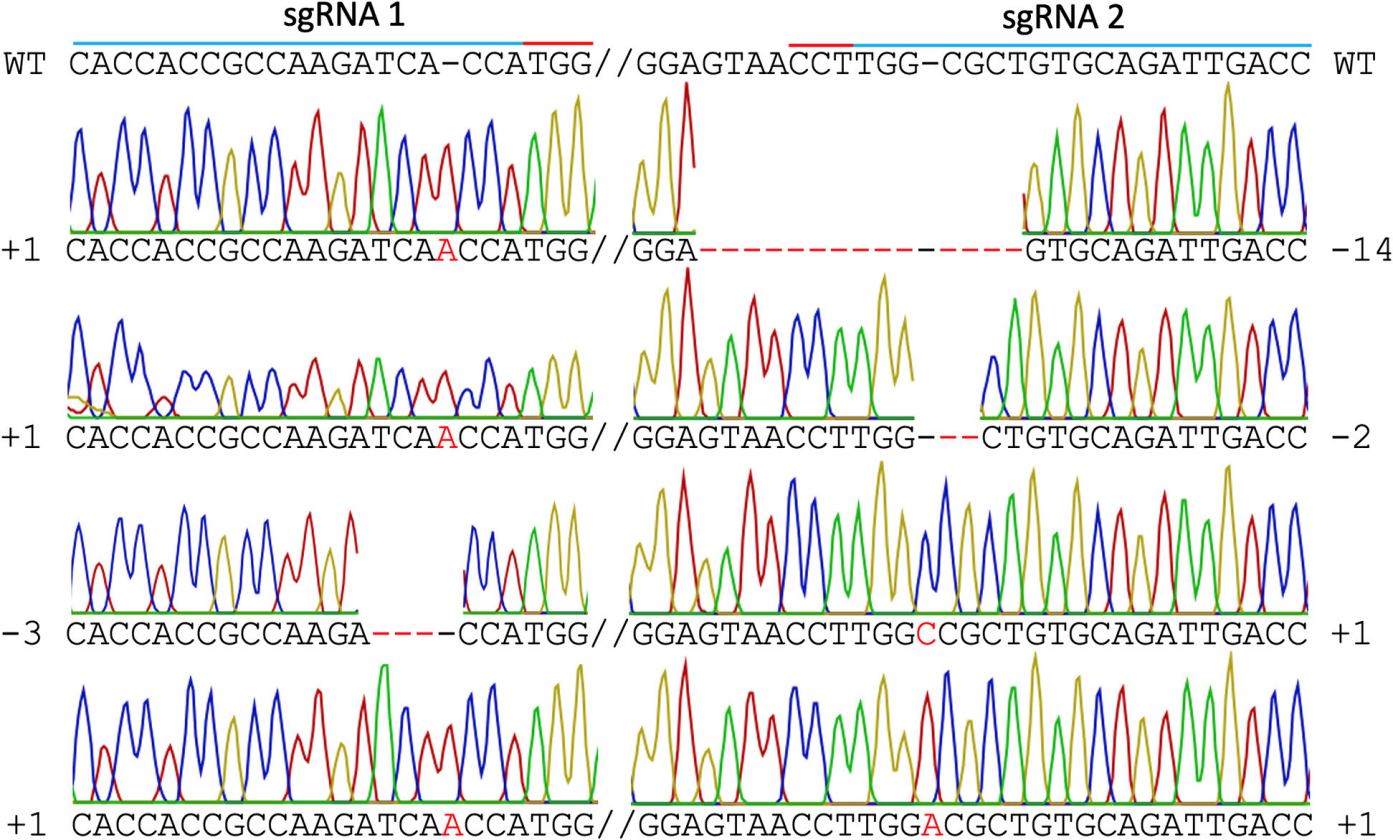
Sanger sequencing of line HY1. Different types of edits are displayed. The blue line indicates both sgRNA target sites, the red line indicates PAM sites, the red font indicates insertions, and the red dashed line indicates deletions. WT = wild type.

In mutant line HY1 (Figure 3B), 59 unique reads were identified from 175 cloned *MgCh* PCR amplicons, representing individual *MgCh* copies/alleles. In total, 49 of the 59 copies/alleles from HY1 were edited (83.1%), and 10 were not edited (16.9%). Among the 49 edited copies/alleles, 27 copies/alleles (45.8%) only displayed edits at the target site of sgRNA1 (PAM1), 3 (5.1%) only displayed edits at the target site of sgRNA2 (PAM2), and 19 (32.2%) displayed edits at both target sites (Figure 5, Supplementary Tables 2, 3). Among the 49 edited copies/alleles, 40 (67.7% of all unique reads) carried frame-shift mutations, and 9 (15.3% of all unique reads) displayed a single amino acid deletion or in-frame isoform (Supplementary Tables 2, 4). Indel analyses revealed that the most dominant deletions were 2–3 nt long, the longest deletion was 14 nt, and the detected insertions were all 1 nt long (Figure 5, Supplementary Tables 2–4).

### SPAD Chlorophyll Meter Analyses to Quantify Leaf Greenness in Mutant *MgCh* Lines

Leaf greenness from mutant and WT plants was determined using the SPAD chlorophyll meter (Minolta SPAD-502, Konica-Minolta). Two lines were derived from incubation at 28°C following gene transfer (non-heat-treated green lines NG1 and NG3) that displayed SPAD values comparable to the WT leaves which was detected as 43.39 SPAD units (Table 2). Line NG2 displayed a 9% lower SPAD value than WT, but its greenness was not visibly distinguishable from WT (Figure 3C). Therefore, mutant plants derived from incubation at 28°C were considered to be lacking a chlorophyll-depletion phenotype. The green lines originating from the heat-treated callus (37°C, HG1, HG2, and HG3) displayed SPAD values of 30.63, 34.7, and 31.07, respectively. This indicated a 20 to 29% reduction of chlorophyll compared to WT, although this was not visibly distinguishable from WT (Table 2; Figure 3C). The yellow lines (HY1, HY2, and HY3) originating from the heat-treated callus displayed SPAD values of 5.5, 6.63, and 12.53, respectively. This indicates a 71 to 87% reduction in chlorophyll compared to WT and was visibly distinguishable from the WT, NG, and HG lines (Table 2, Figure 3C).

**Table 2:** Evaluation of leaf pigmentation of edited lines using a SPAD meter.

**Line**	**Heat (37°C)**	**SPAD value**
WT	-	43.93 ± 0.71
NG1	No	43.87 ± 1.85
NG2	No	40.03 ± 2.55
NG3	No	43.27 ± 0.95
HG1	Yes	30.63 ± 1.37
HG2	Yes	34.70 ± 2.27
HG3	Yes	31.07 ± 1.26
HY1	Yes	5.50 ± 1.08
HY2	Yes	6.63 ± 1.21
HY3	Yes	12.53 ± 1.3
LSD		3.8

*Chlorophyll pigmentation values of edited lines (NG = non-treated green, HG = heat-treated green, HY = heat-treated yellow) as compared to non-modified sugarcane cultivar CP88-1762 (WT). Values were generated by SPAD chlorophyll meter from greenhouse-grown plants. The values following the means are standard deviation. Lines = 10; n = 3; LSD = least significant difference (3.8); p < 0.01*.

### Quantifying Multiallelic Co-editing Efficiency of *MgCh* With Next-Generation Sequencing

Next-generation sequencing data showed that the observed level of chlorophyll depletion largely corresponded to the proportion of edited reads. The exception to this was line HG3, which had only 4.4–4.8% of the reads edited at sgRNA target site 2 or 1, respectively, and displayed a 28% reduction of chlorophyll (Tables 2, 3). *MgCh* reads aligning to the sgRNA1 target site displayed editing efficiencies ranging from 6.9 to 20.8% for NG lines, 4.8 to 28.8% for HG lines, and 42 to 82.3% for HY lines (Table 3). The editing efficiencies at the sgRNA2 target ranged from 5.7 to 8.7% for the NG lines, 4.4 to 29% for the HG lines, and 18.7 to 19.7% for the HY lines (Table 3). Taking the highest editing efficiency for each line at either the sgRNA1 or sgRNA2 target sites, NG lines displayed a range from 6.9 to 20.8%, HG lines from 4.8 to 29%, and HY lines from 42 to 82.3% of the *MgCh* reads as edited NGS reads (Table 3). The most common edit detected at the sgRNA1 target site was insertion, with an average of 24.3% of the total events across all of the lines. The most frequent editing event at sgRNA2 target site was a combination of substitution and insertion, with 8.6% of the total events across all of the lines (Table 3).

**Table 3:** Summary of editing events detected by next-generation sequencing in the 20-nt sgRNA target sites.

**Line**	**Type of edits in percent of NGS reads from target amplicon**															
	**sgRNA1 Target Site**								**sgRNA2 Target Site**							
	**Total edits**	**S**	**I**	**SI**	**D**	**SD**	**ID**	**SID**	**Total edits**	**S**	**I**	**SI**	**D**	**SD**	**ID**	**SID**
NG1	6.9	4.2	1.8	0.9	0.0	0.0	0.0	0.0	5.7	3.7	0.2	1.5	0.2	0.0	0.1	0.0
NG2	12.4	3.9	3.7	0.8	3.9	0.1	0.0	0.0	7.6	3.7	0.3	3.1	0.5	0.0	0.0	0.0
NG3	20.8	3.5	16.3	1.0	0.0	0.0	0.0	0.0	8.7	3.7	0.5	4.2	0.3	0.0	0.0	0.0
HG1	28.8	2.9	15.7	1.1	0.4	0.1	8.4	0.2	29	2.5	0.2	25.9	0.4	0.0	0.0	0.0
HG2	22.5	3.5	11.9	0.9	0.3	0.0	5.8	0.1	24.3	3.0	0.2	20.8	0.3	0.0	0.0	0.0
HG3	4.8	4.3	0.4	0.1	0.0	0.0	0.0	0.0	4.4	3.7	0.2	0.3	0.1	0.0	0.1	0.0
HY1	78.1	1.3	64.3	12.5	0.0	0.0	0.0	0.0	19.7	3.4	0.2	4.4	11.7	0.0	0.0	0.0
HY2	82.3	1.0	67.9	13.4	0.0	0.0	0.0	0.0	18.7	3.2	0.2	2.7	12.5	0.0	0.1	0.0
HY3	42	3.3	36.9	1.8	0.0	0.0	0.0	0.0	19.4	3.4	1.3	14.6	0.1	0.0	0.0	0.0
Mean	33.1	3.1	24.3	3.6	0.5	0.0	1.6	0.0	15.3	3.4	0.4	8.6	2.9	0.0	0.0	0.0
WT	4.8	4.6	0.1	0.1	0.0	0.0	0.0	0.0	4.0	3.5	0.2	0.1	0.1	0.0	0.1	0.0

*Values represent edited reads in percent of total reads that align to the target amplicon. Totals are the sums of all types of edits. The values at the sgRNA2 target site include the compilation of three allelic variants at this site. S = substitution, SI = substitution + insertion, D = deletion; SD = substitution + deletion, ID = insertion + deletion; SID = substitution + insertion + deletion; NG = not heat-treated, green; HG = heat-treated, green; HY = heat-treated, yellow; WT = unmodified sugarcane cultivar CP88-1762*.

On average of all the edited lines, 33.1 or 15.3% of all NGS reads, that aligned to sgRNA1 or sgRNA2 displayed edits, respectively (Table 3). The sgRNA1 target site was located in a highly conserved region of *MgCh* with no sequence variants in WT. sgRNA2 was designed to target a site where three sequence variants were present in WT, with 0, 1, or 2 nt mismatches against the corresponding sgRNA. The editing efficiency was highest at the variant, with no mismatches to the sgRNA, reaching up to 28% of the *MgCh* reads in line HG1. Both variants, displayed low but detectable editing efficiencies with 1 or 2 mismatches (Supplementary Table 5). The NGS reads were also analyzed to detect long insertions and long deletions at both sgRNA1 and sgRNA2 target sites. Long insertions were detected only at sgRNA site 1, in up to 6.5% of the *MgCh* reads as edited NGS reads for line HG1 (Supplementary Table 6). In lines with significant more long insertions than the wild type, the average length of long insertions ranged from 6.8 bp in NG1 to 9.4 bp in HG1 (Supplementary Table 6). Long deletions at sgRNA1 target site were detected in <0.1% of the NGS reads. At sgRNA2 target site long deletions were detected in 0.5% (HG3) to 19.4% (HY2) of the *MgCh* reads. In lines with significant more long deletions than the wild type, the average length of long deletions ranged from 5.9 bp in HY3 to 14.3 in HY2 (Supplementary Table 6).

## Discussion

Polyploidy is a common challenge in functional genomics and genetic improvement for many important crops. Sugarcane is an interspecific hybrid with a highly polyploid genome (x = 10 − 13; 2n = 100 − 130) typically containing 10 or more homo(eo)logs at each locus (Le Cunff et al., 2008). This high level of genetic redundancy requires very efficient co-editing of multiple copies/alleles for the generation of knockout or knockdown mutant phenotypes. Here, we describe CRISPR/Cas9-mediated targeted mutagenesis of the magnesium chelatase gene (*MgCh*), which is a high-copy gene in sugarcane. Co-editing of up to 49 of the 59 detected copies/alleles was confirmed by Sanger sequencing. Of the 30 transgenic lines harboring the *MgCh* gene editing (MGE) construct, six were confirmed to have targeted mutations for an editing efficiency of 20%. Three events with the co-editing of the majority of the *MgCh* copies/alleles displayed severe chlorophyll depletion, which was visibly scorable as the plantlets regenerated from tissue culture.

In hexaploidy wheat, a tri-genome targeted sgRNA to the *PDS* gene was co-introduced with Cas9 in 38 independent transgenic lines, but no photobleaching phenotype was identified. Only 2 of the 38 transgenic wheat lines displayed targeted mutagenesis (editing efficiency of 5%), and none of them displayed co-editing of multiple copies/alleles (co-editing efficiency of 0%) (Howells et al., 2018). By contrast, the diploid barley displayed an editing efficiency that was three times higher than wheat, with the same construct. However, in barley, only chimeric events were identified, displaying the photo-bleaching phenotype in sections of the leaves that were associated with progressive somatic edits (Howells et al., 2018). Generally, short indels that include insertions, substitutions, and deletions are highly reported events in gene editing in the polyploid plant genomes (Naim et al., 2018; Shan et al., 2018; Wang et al., 2020; Wolabu et al., 2020).

To the best of our knowledge, this report is the first to describe the targeted mutagenesis of *MgCh* in plants. Naturally occurring mutations in the *MgCh* gene have previously been reported to cause impaired chlorophyll biosynthesis and thus to result in phenotypes with light green or yellow foliage (Campbell et al., 2014). RNA interference of *MgCh* in tobacco and peach results in light green phenotypes with significantly reduced chlorophyll contents (Papenbrock et al., 2000). By contrast to *MgCh* mutants, PDS mutants display impaired chlorophyll, carotenoid, and gibberellin biosynthesis resulting in dwarf or albino plantlets in both biallelic, homozygous, and biallelic heterozygous events (Qin et al., 2007). Albino plants may also be caused by somaclonal variation, and the dwarfing resulting from PDS suppression makes tissue collection to confirm molecular analyses more challenging. By contrast, Mg-chelatase-impaired natural mutants have been described with yields similar to (Slattery et al., 2017) or higher than WT plants (Pettigrew et al., 1989) despite 50% reduced chlorophyll content. The latter is associated with increased light penetration into the canopy, causing an increase in the CO_2_ exchange rate there. The former results in a reduced nitrogen requirement. Therefore, manipulating chlorophyll content has been proposed as a strategy for improving canopy-level photosynthesis or nitrogen use efficiency under the dense canopies of tall biomass plants such as sugarcane (Kirst et al., 2017; Walker et al., 2018).

In this study, we demonstrated that the targeted mutagenesis of magnesium chelatase with CRISPR/Cas9 provides a rapidly scorable phenotype without leading to obvious growth retardation. Notably, the level of chlorophyll depletion was predictive of the extent of multiallelic co-editing of *MgCh*, which enables a rapid readout of the editing outcome.

The efficiencies of Cas9- and Cas12a-mediated mutagenesis can be elevated by the heat treatment of the callus following the delivery of editing reagents, as previously reported for *Arabidopsis*, rice, maize, and wheat (LeBlanc et al., 2018; Malzahn et al., 2019; Milner et al., 2020). Therefore, we deployed an MGE-based rapid readout system to compare co-editing efficiencies in response to heat treatment (37°C) or standard incubation temperature (28°C) for sugarcane callus. Three transgenic lines emerging from the heat treatment and none of the transgenic lines that regenerate under standard incubation temperatures displayed a phenotype with visible chlorophyll depletion. Genotyping with assaying restriction enzyme loss in the sgRNA target region, Sanger sequencing, and next-generation sequencing revealed a total of three mutant lines per 10 shots from callus regeneration under standard incubation temperatures and six mutant lines per 10 shots from heat-treated tissue. The events which were visibly distinguishable from WT due to severe chlorophyll depletion following heat treatment displayed editing of more than 40% of the *MgCh* NGS target amplicon reads. The events from standard temperature treatment displayed an editing rate of 6.9–20.8% of the *MgCh* NGS target amplicon reads without displaying a phenotype that could be visibly distinguished from WT. This suggested that heat treatment elevated the editing frequency 2-fold and drastically promoted the co-editing of multiple alleles to create a phenotype that was visibly distinguishable from WT. Efficient co-editing of multiple copies/alleles is of major importance for the generation of a distinct mutant phenotype in vegetatively propagated polyploid crops such as sugarcane and potato, where the combination of mutant alleles via sexual hybridization would disrupt the highly heterozygous, elite cultivar.

Multiallelic gene editing via CRISPR/Cas9 has been reported in several polyploid crops. In studies of both tetraploid potato and tetraploid switchgrass, only 2.0% of the T_0_ transgenic plants displayed co-mutation of all four targeted alleles of the granule-bound starch synthase gene or phosphoglycerate mutase gene (Andersson et al., 2017; Liu et al., 2018). Using polycistronic delivery for four sgRNAs instead of a single one dramatically elevated the editing efficiency in the tetraploid alfalfa. None of the 339 transgenic lines harboring a single sgRNA or Cas9 displayed tetra-allelic editing or the desired stay-green phenotype. By contrast, seven lines were confirmed with co-editing of all four alleles of the stay-green gene (MsSGR) in T_0_ from 492 transgenic lines (Wolabu et al., 2020).

Co-delivery of two sgRNA instead of a single sgRNA also elevated the co-editing efficiency in this study. Analyses of unique reads following the Sanger sequencing of cloned PCR amplicons of line HY1 revealed that 45.8% of the reads were edited only at sgRNA site 1, 5.1% were edited only at sgRNA site 2, and 32.2% were edited at both sites. Both Sanger and next-generation sequencing analyses suggested that functional knockouts are mostly composed of short insertions and short deletions resulting in out of frame mutations. For example, in mutant line HY1, with severe depletion of chlorophyll, 67.8% of the alleles were out of frame.

To exploit the reduction in chlorophyll content for elevating the canopy level photosynthesis, it may be desirable to target a limited number of *MgCh* copies/alleles for mutagenesis. The choice of sgRNAs allows the targeting of specific alleles. Unlike sgRNA1, which was targeted to a highly conserved region with no allelic variants, sgRNA2 was targeted to a region that had three allelic variants with zero, one, or two mismatches to the sgRNA (Supplementary Table 5). Co-delivering both sgRNAs allowed a comparison of the impact of the individual sgRNA on the editing efficiency and the editing of the different allelic variants. Higher editing efficiency was found for all mutants for the target of sgRNA1 (33.1% edited NGS reads) than for sgRNA2 (15.3% edited NGS reads). The allelic variants with SNPs in sgRNA2 target region displayed very few edits and contributed to the overall lower editing efficiency at this site. This suggests that the choice of gRNA and the combination of sgRNAs offers opportunities to tailor the desired co-editing efficiency in sugarcane, similar to what is reported for other polyploid crops (Andersson et al., 2017; Liu et al., 2018; Wolabu et al., 2020).

The described approach will facilitate the establishment of genome-editing protocols for recalcitrant crops and will support important optimizations for the elevation of gene-editing efficiencies, including the evaluation of alternative tissue culture protocols, genome editing reagents, and their delivery.

## Data Availability Statement

The datasets presented in this study can be found in online repositories. The names of the repository/repositories and accession number(s) can be found below: https://www.ncbi.nlm.nih.gov/, Bioproject ID PRJNA704370.

## Author Contributions

FA conceived, designed, and managed the research project. DW and FA designed the sgRNAs. DW and AE validated sgRNAs with *in vitro* assays. CM generated the recombinant DNA constructs. CM and SS carried out the tissue cultures and generated the transgenic sugarcane plants. AE carried out the molecular and phenotypic analyses of the regenerated sugarcane lines. FA and AE wrote the manuscript. All authors read and approved the final manuscript.

## Conflict of Interest

The authors declare that the research was conducted in the absence of any commercial or financial relationships that could be construed as a potential conflict of interest.

## Supplementary Material

Supplementary Figure 1 Sanger sequencing reads exemplifying targeted mutations in different lines. The blue line indicates both sgRNA target sites, the orange line indicates PAM sites, the red font indicates insertions, and the red dashed line indicates deletions. Lines HY2, HY3, NG1, NG2, NG3, HG1, and HG3. HY = heat-treated, yellow; NG = non-heat treated, green; HG = heat treated, green; WT = wild type.

Supplementary Table 1 Primers used in the experiment.

Supplementary Table 2 Summary of edits of mutant line HY1 as detected by Sanger sequencing of cloned PCR amplicons of *MgCh*.

Supplementary Table 3A Genotyping of 59 alleles/variants as detected by Sanger sequencing of cloned PCR amplicons of *MgCh* in mutant line HY1.

Supplementary Table 3B Genotyping of 59 alleles/variants as detected by Sanger sequencing of cloned PCR amplicons of *MgCh* in mutant line HY1.

Supplementary Table 4 Types of edits in *MgCh* gene in line HY1.

Supplementary Table 5 Summary of Next Generation Sequencing revealing the editing frequency at target site for sgRNA2 for 3 different allelic variants of the *MgCh* gene.

Supplementary Table 6 Analysis of NGS reads that did not align to sgRNA target sequence due to long insertions or long deletions.
