# Supplementary figures and images for: Multiallelic, Targeted Mutagenesis of Magnesium Chelatase With CRISPR/Cas9 Provides a Rapidly Scorable Phenotype in Highly Polyploid Sugarcane
Source: Front Genome Ed. 2021 Apr 29;3:654996. doi: 10.3389/fgeed.2021.654996 (PMC8525377; doi:10.3389/fgeed.2021.654996)

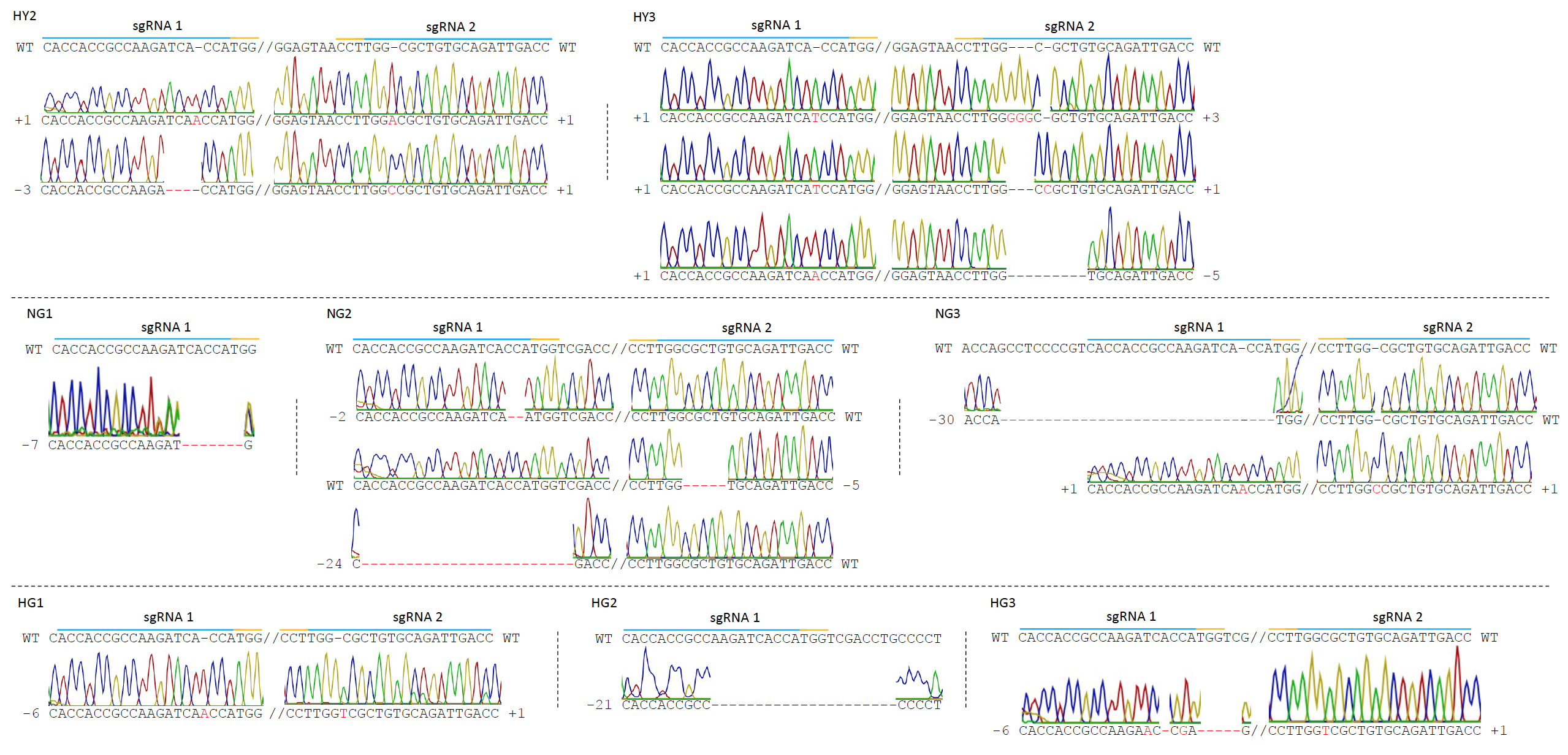

Supplement: Supplementary Figure 1 — Sanger sequencing reads exemplifying targeted mutations in different lines. The blue line indicates both sgRNA target sites, the orange line indicates PAM sites, the red font indicates insertions, and the red dashed line indicates deletions. Lines HY2, HY3, NG1, NG2, NG3, HG1, and HG3. HY = heat-treated, yellow; NG = non-heat treated, green; HG = heat treated, green; WT = wild type. [file Image_1.tiff]
