# Supplementary material for: Multiallelic, Targeted Mutagenesis of Magnesium Chelatase With CRISPR/Cas9 Provides a Rapidly Scorable Phenotype in Highly Polyploid Sugarcane
Source: Front Genome Ed. 2021 Apr 29;3:654996. doi: 10.3389/fgeed.2021.654996 (PMC8525377; doi:10.3389/fgeed.2021.654996)
Supplement: Supplementary Table 1 — Primers used in the experiment. [file Data_Sheet_1.docx]

**Supplementary Table 1: Primers used in the experiment**

| Name | Sequence 5’-3’ |
| --- | --- |
| T7MgCh1F | TAATACGACTCACTATAGGGCACCACCGCCAAGATCACCAGTTTTAGAGCTAGAAATAGCAAGTTAAAATAG |
| T7MgCh2F | TAATACGACTCACTATAGGGTCAATCTGCACAGCGCCAGTTTTAGAGCTAGAAATAGCAAGTTAAAATAG |
| ScaffoldR1 | AAAAAAGCACCGACTCGGTGCCACTTTTTCAAGTTGATAACGGACTAGCCTATTTTAACTTGCTATTTCT |
| T7F | GGATCCTAATACGACTCACTATAG |
| ScaffoldR2 | AAAAAAGCACCGACTCGG |
| C27 | CTCAACGTCATCGACCCCAA |
| C28 | CTTGGCAGCCCTGTTAGTCA |
| sgRNA1F | gcttGCACCACCGCCAAGATCACCA |
| sgRNA1R | aaacTGGTGATCTTGGCGGTGGTGC |
| sgRNA2F | gcttGGTCAATCTGCACAGCGCCA |
| sgRNA2R | aaacTGGCGCTGTGCAGATTGACC |
| C5 | GACATCCGCGTCGTCTTCGGC |
| C9 | CATAAAACTTCTCAATGACGAGCA |
| C31 | GTCCGCGAGCGGGTC |
| C32 | ATATCTTGACGCGGAGGTCAT |

**Supplementary Table 2: Summary of edits of mutant line HY1 as detected by Sanger sequencing of cloned PCR amplicons of *MgCh***

| **Mutant Line** | **Edited only in sgRNA1 target** | **Edited only in sgRNA2 target** | **Co-edited in sgRNA1+2 targets** | **Total reads with edits** | **Non-edited reads** | **In-frame reads** | **Out of-frame reads** |
| --- | --- | --- | --- | --- | --- | --- | --- |
| HY1 | 27 (45.8%) | 3 (5.1%) | 19 (32.2%) | (49/59) (83.1%) | 10 (16.9%) | 19 (32.2%) | 40 (67.8%) |

In line HY1, 59 unique reads were detected by Sanger sequencing of cloned PCR amplicons based on combinations of single nucleotide polymorphisms outside of the sgRNA target regions. These represent independent copies/alleles of *MgCh*. This table summarizes the more detailed Supplementary Table 3 a & b. Data represent only the distribution of unique reads detected during the analysis.

**Supplementary Table 3A: Genotyping of 59 alleles/variants as detected by Sanger sequencing of cloned PCR amplicons of *MgCh* in mutant line HY1**

|  |  |  |  |  |  |  |  |  |  | **P** | **A** | **M** |  |  |  |  |  |  |  |  |  |  |  |  |  |  |  |  |  |  | **P** | **A** | **M** |  |  |  |  |  |  |  |  |  |  |  |  |  |  |  |  |  |
| --- | --- | --- | --- | --- | --- | --- | --- | --- | --- | --- | --- | --- | --- | --- | --- | --- | --- | --- | --- | --- | --- | --- | --- | --- | --- | --- | --- | --- | --- | --- | --- | --- | --- | --- | --- | --- | --- | --- | --- | --- | --- | --- | --- | --- | --- | --- | --- | --- | --- | --- |
|  | 744 | 745 | 746 | 747 |  | 748 | 749 | - | 750 | **751** | **752** | **753** | 822 | 840 | 868 | 912 | 945 | 990 | 1116 | 1150 | 1083 | 1161 | 1157 | 1170 | 1176 | 1213 | 1217 | 1218 | 1219 | 1220 | **1221** | **1222** | **1223** | 1224 | 1225 | 1226 | - | 1227 | - | 1228 | 1229 | 1230 | 1240 | 1283 | 1337 | 1338 | 1346 | 1364 | 1365 | 1376 |
| WT | A | T | C | A | - | C | C |  | A | **T** | **G** | **G** | C | C | A | T | C | T | C | G | T | C | T | C | G | C | G | T | A | A | **C** | **C** | **T** | T | G | G | - | C | - | G | C | T | T | G | G | T | G | C | G | G |
| 1 |  |  |  |  | - | **-** | **-** |  | **-** |  |  |  |  |  |  |  | T |  |  |  |  |  |  |  |  |  |  |  |  |  |  |  |  |  |  | **-** | - | **-** |  |  |  |  |  |  |  |  | A |  |  |  |
| 2 |  |  |  |  | - | **-** | **-** |  | **-** |  |  |  |  |  |  |  | T |  |  | A |  | T |  |  |  |  |  |  |  |  |  |  |  |  |  |  | - |  |  |  |  |  |  |  |  | T |  |  |  |  |
| 3 |  | **-** | **-** | **-** | - |  |  |  |  |  |  |  | G |  | C |  |  |  |  | A |  | T |  |  |  |  |  |  |  |  |  |  |  |  |  |  | - |  |  |  |  |  |  |  |  | T |  | T |  |  |
| 4 |  |  |  |  | A |  |  |  |  |  |  |  | G |  | C |  |  |  |  |  |  |  |  |  |  |  |  |  |  |  |  |  |  |  |  | **-** | - | **-** |  |  |  |  |  |  |  |  | A |  |  |  |
| 5 |  |  |  |  | A |  |  |  |  |  |  |  |  |  |  |  | G |  |  |  |  |  |  |  |  |  |  |  |  |  |  |  |  |  |  | **-** | - | **-** |  |  |  |  |  |  |  |  | A |  |  |  |
| 6 |  |  |  |  | A |  |  |  |  |  |  |  |  | G |  |  | G |  |  |  |  |  |  |  |  |  |  |  |  |  |  |  |  |  |  | **-** | - | **-** |  |  |  |  |  |  |  |  | A |  |  |  |
| 7 |  | **-** | **-** | **-** | - |  |  |  |  |  |  |  |  |  |  |  | G |  |  |  |  |  |  |  |  |  |  |  |  |  |  |  |  |  |  | **-** | - | **-** |  |  |  |  |  |  |  |  | A |  |  |  |
| 8 |  |  |  |  | A |  |  |  |  |  |  |  |  | G |  |  | G |  |  |  |  |  |  |  |  |  |  |  |  |  |  |  |  |  |  | **-** | - | **-** |  |  |  |  |  |  |  |  |  |  |  |  |
| 9 |  |  |  |  | - | **-** | **-** |  | **-** |  |  |  |  |  |  |  | T |  |  |  | G |  | C |  | A |  |  |  |  |  |  |  |  |  |  |  | - | T |  |  |  |  |  | A | A |  |  |  |  | A |
| 10 |  |  |  |  | - | **-** | **-** |  | **-** |  |  |  |  |  |  |  | T |  |  |  | G |  |  |  | A |  |  |  |  |  |  |  |  |  |  |  | - | T |  |  |  |  |  | A | A |  |  |  |  | A |
| 11 |  |  |  |  | - | **-** | **-** |  | **-** |  |  |  |  |  |  |  | T |  |  |  | G |  |  |  | A |  |  |  |  |  |  |  |  |  |  |  | - | T |  |  |  |  |  | A | A |  |  |  |  |  |
| 12 |  |  |  |  | - | **-** | **-** |  | **-** |  |  |  |  |  |  |  | T |  |  |  | G |  |  |  | A |  |  |  |  |  |  |  |  |  |  |  | - | T |  |  |  |  |  | A |  | T |  | T |  |  |
| 13 |  |  |  |  | - | **-** | **-** |  | **-** |  |  |  |  |  |  |  |  |  |  |  | G |  |  |  | A |  |  |  |  |  |  |  |  |  |  |  | - | T |  |  |  |  |  | A |  | T |  | T |  |  |
| 14 |  |  |  |  | A |  |  |  |  |  |  |  |  |  |  |  |  |  |  |  |  |  |  |  | A |  |  |  |  |  |  |  |  |  |  |  | - | T |  |  |  |  |  | A | A |  |  |  |  | A |
| 15 |  |  |  |  | A |  |  |  |  |  |  |  |  |  |  |  |  |  |  |  | G |  |  |  | A |  |  |  |  |  |  |  |  |  |  |  | - | T |  |  |  |  |  | A | A |  |  |  |  | A |
| 16 |  |  |  |  | A |  |  |  |  |  |  |  |  |  |  |  | T |  |  |  | G |  |  |  | A |  |  |  |  |  |  |  |  |  |  |  | - | T |  |  |  |  |  | A | A |  |  |  |  | A |
| 17 |  |  |  |  |  |  |  | C |  |  |  |  |  |  |  |  | G |  |  |  |  | T |  |  | A |  |  |  |  |  |  |  |  |  |  |  | - | T |  |  |  |  |  | A | A |  |  |  |  | A |
| 18 |  |  |  |  | A |  |  |  |  |  |  |  |  |  |  |  | G |  |  |  | G |  |  |  | A |  |  |  |  |  |  |  |  |  |  |  | - | T |  |  |  |  |  |  |  |  | A |  |  |  |
| 19 |  |  |  |  | A |  |  |  |  |  |  |  |  |  |  |  |  |  |  |  | G |  |  |  | A | A |  |  |  |  |  |  |  |  |  |  | - | T |  |  |  |  | C |  |  | T |  |  |  |  |
| 20 |  |  |  |  |  |  |  | C |  |  |  |  |  |  |  |  |  |  |  |  | G |  |  |  | A | A |  |  |  |  |  |  |  |  |  |  | - | T |  |  |  |  | C |  |  | T |  |  |  |  |
| 21 |  |  |  |  | A |  |  |  |  |  |  |  |  |  |  | C |  |  |  |  | G |  |  |  | A | A |  |  |  |  |  |  |  |  |  |  | - | T |  |  |  |  | C |  |  | T |  |  |  |  |
| 22 |  |  |  |  | A |  |  |  |  |  |  |  |  |  |  |  |  |  |  |  | G |  |  |  | A | A |  |  |  |  |  |  |  |  |  |  | - | T |  |  |  |  | C |  |  | T |  | T |  |  |
| 23 |  |  |  |  | A |  |  |  |  |  |  |  |  |  |  |  | G |  |  |  |  | T |  |  |  |  |  |  |  |  |  |  |  |  |  |  | - |  |  |  |  |  |  |  |  | T |  |  |  |  |
| 24 |  |  |  |  | A |  |  |  |  |  |  |  |  |  |  |  |  |  |  |  |  |  |  |  |  |  |  |  |  |  |  |  |  |  |  |  | - |  |  |  |  |  |  |  | A | T |  |  |  |  |
| 25 |  |  |  |  | A |  |  |  |  |  |  |  |  |  |  |  |  |  |  |  |  |  |  |  |  |  |  |  |  |  |  |  |  |  |  |  | - |  |  |  |  |  |  |  |  | T |  |  |  |  |
| 26 |  |  |  |  | A |  |  |  |  |  |  |  |  |  |  |  | G |  |  |  |  |  |  |  |  |  |  |  |  |  |  |  |  |  |  |  | - |  |  |  |  |  |  |  |  | T |  |  |  |  |
| 27 |  |  |  |  |  |  |  | C |  |  |  |  |  |  |  |  | G |  |  |  |  | T |  |  |  | A |  |  |  |  |  |  |  |  |  |  | - | T |  |  |  |  | C |  |  | T |  |  |  |  |
| 28 |  | **-** | **-** | **-** | - |  |  |  |  |  |  |  |  |  |  | C |  |  |  | A |  | T |  |  |  |  |  |  |  |  |  |  |  |  |  |  | - |  |  |  |  |  |  |  |  | T |  |  |  |  |
| 29 |  |  |  |  | A |  |  |  |  |  |  |  |  |  | C |  | C |  |  |  |  |  |  |  |  |  |  |  |  |  |  |  |  |  |  |  | - |  |  |  |  |  |  |  |  |  |  |  |  |  |
| 30 |  |  |  |  | A |  |  |  |  |  |  |  |  | G |  |  | G |  |  |  |  |  |  |  |  |  |  |  |  |  |  |  |  |  |  |  | - |  |  |  |  |  |  |  | A | T |  |  |  |  |
| 31 |  |  |  |  | A |  |  |  |  |  |  |  |  |  |  |  |  |  |  |  |  |  |  |  |  |  |  |  |  |  |  |  |  |  |  |  | - |  |  |  |  |  |  |  | A | T |  |  |  | A |
| 32 |  |  |  |  | A |  |  |  |  |  |  |  |  |  |  | C |  |  |  | A |  | T |  |  |  |  |  |  |  |  |  |  |  |  |  |  | - |  |  |  |  |  |  |  |  | T |  |  |  |  |
| 33 |  |  |  |  | A |  |  |  |  |  |  |  |  |  |  | C |  |  |  | A |  | T |  |  |  |  | **-** | **-** | **-** | **-** | **-** | **-** | **-** | **-** | **-** | **-** | - | **-** |  | **-** | **-** | **-** |  |  |  | T |  |  |  |  |
| 34 |  |  |  |  | A |  |  |  |  |  |  |  |  |  |  |  |  |  |  |  |  |  |  |  |  |  | **-** | **-** | **-** | **-** | **-** | **-** | **-** | **-** | **-** | **-** | - | **-** |  | **-** | **-** | **-** |  |  |  | T |  |  |  |  |

**Supplementary Table 3B: Genotyping of 59 alleles/variants as detected by Sanger sequencing of cloned PCR amplicons of *MgCh* in mutant line HY1**

**– continued –**

|  |  |  |  |  |  |  |  |  |  | **P** | **A** | **M** |  |  |  |  |  |  |  |  |  |  |  |  |  |  |  |  |  |  | **P** | **A** | **M** |  |  |  |  |  |  |  |  |  |  |  |  |  |  |  |  |  |
| --- | --- | --- | --- | --- | --- | --- | --- | --- | --- | --- | --- | --- | --- | --- | --- | --- | --- | --- | --- | --- | --- | --- | --- | --- | --- | --- | --- | --- | --- | --- | --- | --- | --- | --- | --- | --- | --- | --- | --- | --- | --- | --- | --- | --- | --- | --- | --- | --- | --- | --- |
|  | 744 | 745 | 746 | 747 | - | 748 | 749 |  | 750 | **752** | **752** | **754** | 822 | 840 | 868 | 912 | 945 | 990 | 1116 | 1150 | 1083 | 1161 | 1157 | 1170 | 1176 | 1213 | 1217 | 1218 | 1219 | 1220 | **1221** | **1222** | **1223** | 1224 | 1225 | 1226 | - | 1227 | - | 1228 | 1229 | 1230 | 1240 | 1283 | 1337 | 1338 | 1346 | 1364 | 1365 | 1376 |
| WT | A | T | C | A |  | C | C |  | A | **T** | **G** | **G** | C | C | A | T | C | T | C | G | T | C | T | C | G | C | G | T | A | A | **C** | **C** | **T** | T | G | G | - | C | - | G | C | T | T | G | G | T | G | C | G | G |
| 35 |  |  |  |  | A |  |  |  |  |  |  |  |  |  |  | C |  |  |  | A |  | T |  |  |  |  | **-** | **-** | **-** | **-** | **-** | **-** | **-** | **-** | **-** | **-** | - | **-** |  | **-** | **-** | **-** |  |  |  | T |  |  | A |  |
| 36 |  |  |  |  |  |  |  |  |  |  |  |  |  |  |  | C |  |  |  | A |  | T |  |  |  |  |  |  |  |  |  |  |  |  |  |  | C |  |  |  |  |  |  |  |  | T |  | T |  |  |
| 37 |  |  |  |  |  |  |  |  |  |  |  |  | G |  | C |  |  |  |  |  |  |  |  |  |  |  |  |  |  |  |  |  |  |  |  | **-** | - | **-** |  |  |  |  |  |  |  |  | A |  |  |  |
| 38 |  |  |  |  |  |  |  |  |  |  |  |  |  |  |  | C |  |  |  | A |  | T |  |  |  |  | **-** | **-** | **-** | **-** | **-** | **-** | **-** | **-** | **-** | **-** | - | **-** |  | **-** | **-** | **-** |  |  |  | T |  |  |  |  |
| 39 |  |  |  |  | A |  |  |  |  |  |  |  |  |  |  |  | G |  |  |  |  | T |  |  |  |  |  |  |  |  |  |  |  |  |  | **-** | - | **-** |  |  |  |  |  |  |  |  | A |  |  |  |
| 40 |  |  |  |  | A |  |  |  |  |  |  |  |  |  |  |  | G |  |  |  |  | T |  | T |  |  |  |  |  |  |  |  |  |  |  |  | **T** |  |  |  |  |  |  |  |  | T |  | T |  |  |
| 41 |  |  |  |  | A |  |  |  |  |  |  |  |  |  |  |  |  |  |  |  | G |  |  |  |  |  |  |  |  |  |  |  |  |  |  |  | **A** |  |  |  |  |  |  |  | A | T |  |  |  |  |
| 42 |  |  |  |  | A |  |  |  |  |  |  |  |  |  |  |  | G |  |  |  |  | T |  |  |  |  |  |  |  |  |  |  |  |  |  |  | **C** |  |  |  |  |  |  |  |  | T |  | T |  |  |
| 43 |  |  |  |  | A |  |  |  |  |  |  |  |  |  |  |  | G |  |  |  |  | T |  |  |  |  |  |  |  |  |  |  |  |  |  |  | **A** |  |  |  |  |  |  |  |  | T |  | T |  |  |
| 44 |  |  |  |  | A |  |  |  |  |  |  |  |  |  |  | C | G |  |  |  |  | T |  |  |  |  |  |  |  |  |  |  |  |  |  |  | **A** |  |  |  |  |  |  |  |  | T |  | T |  |  |
| 45 |  |  |  |  |  |  |  | C |  |  |  |  |  |  |  |  | G |  |  |  |  | T |  |  |  |  |  |  |  |  |  |  |  |  |  |  | **A** |  |  |  |  |  |  |  |  | T |  | T |  |  |
| 46 |  |  |  |  |  |  |  | C |  |  |  |  |  |  |  |  |  |  |  |  |  | T |  |  |  |  |  |  |  |  |  |  |  |  |  |  | **A** |  |  |  |  |  |  |  |  | T |  | T |  |  |
| 47 |  | **-** | **-** | **-** | - |  |  |  |  |  |  |  |  |  |  |  |  |  | T |  |  |  |  |  |  |  |  |  |  |  |  |  |  |  |  |  | **C** |  |  |  |  |  |  |  |  |  |  | T |  |  |
| 48 |  | **-** | **-** | **-** | - |  |  |  |  |  |  |  |  |  |  |  | C |  | T |  |  |  |  |  |  |  |  |  |  |  |  |  |  |  |  |  | - |  |  |  |  |  |  |  |  |  |  |  |  |  |
| 49 |  | **-** | **-** | **-** | - |  |  |  |  |  |  |  |  |  |  |  |  |  |  |  |  |  |  |  |  |  |  |  |  |  |  |  |  |  |  | - | - | - |  |  |  |  |  |  |  |  |  |  |  |  |
| 50 |  |  |  |  |  |  |  |  |  |  |  |  | G |  | C |  |  |  |  |  |  |  |  |  |  |  |  |  |  |  |  |  |  |  |  |  | - |  |  |  |  |  |  | A | A |  |  |  |  | A |
| 51 |  |  |  |  |  |  |  |  |  |  |  |  |  |  |  | C |  |  |  | A |  |  |  |  | A | A |  |  |  |  |  |  |  |  |  |  | - | T |  |  |  |  | C |  |  | T |  |  |  |  |
| 52 |  |  |  |  |  |  |  |  |  |  |  |  | G |  | C |  | T |  |  |  | G |  |  |  | A |  |  |  |  |  |  |  |  |  |  |  | - | T |  |  |  |  |  | A | A |  |  |  |  | A |
| 53 |  |  |  |  |  |  |  |  |  |  |  |  | G |  | C |  |  |  |  |  |  |  |  |  |  |  |  |  |  |  |  |  |  |  |  |  | - |  |  |  |  |  |  |  |  |  |  |  |  |  |
| 54 |  |  |  |  |  |  |  |  |  |  |  |  |  |  |  | C |  |  |  | A |  | T |  |  |  |  |  |  |  |  |  |  |  |  |  |  | - |  |  |  |  |  |  |  |  | T |  |  |  |  |
| 55 |  |  |  |  |  |  |  |  |  |  |  |  |  |  |  | C |  |  |  | A |  | T |  |  |  |  |  |  |  |  |  |  |  |  |  |  | - |  |  |  |  |  |  |  |  | T |  |  |  |  |
| 56 |  |  |  |  |  |  |  |  |  |  |  |  | G |  | C |  |  |  |  |  | G |  |  |  | A | A |  |  |  |  |  |  |  |  |  |  | - | T |  |  |  |  |  |  |  | T |  |  |  |  |
| 57 |  |  |  |  |  |  |  |  |  |  |  |  | G |  | C |  |  |  |  | A |  | T |  |  |  |  |  |  |  |  |  |  |  |  |  |  | - |  |  |  |  |  |  |  |  | T |  |  |  |  |
| 58 |  |  |  |  |  |  |  |  |  |  |  |  | G |  | C |  |  |  |  |  |  |  |  |  |  |  |  |  |  |  |  |  |  |  |  |  | - |  |  |  |  |  |  |  | A | T |  |  |  |  |
| 59 |  |  |  |  |  |  |  |  |  |  |  |  | G |  | C |  |  |  |  |  |  |  |  |  |  |  |  |  |  |  |  |  |  |  |  |  | - |  |  |  |  |  |  |  |  | T |  |  |  |  |

Analysis of *MgCh* mutant line HY1 by Sanger sequencing. There are 59 unique reads detected by Sanger sequencing based on SNP combinations. Orange font indicates PAM1 sequence. Purple font indicates PAM2 sequence. WT is wild type sequence obtained from the monoploid sugarcane genome database under accession (Sh_016G12_contig-1_g000020). Black letters in reads 1 to 59 represent the SNPs detected in allelic variants/copies. Red font indicates reads with co-editing at the target site of sgRNA1 + 2. Green font indicates reads that display editing events only in the sgRNA 1 target region. Blue font indicates reads that display editing events only in the sgRNA 2 target region. Sequencing reads were aligned with the multiple sequence alignment tool CLUSTALW and redundant reads were all removed, to show only unique reads based on unique SNP combinations.

# Supplementary Table 4: Types of edits in *MgCh* gene in line HY1

| HY1 Variants | Non-synonymous mutation | Frame shift and protein truncation | Amino acid deletion | In frame |
| --- | --- | --- | --- | --- |
| 3D:2D:1 | - | + | + | - |
| 3D:2 | - | - | + | + |
| 3D:3 | - | - | + | + |
| 1I:2D:4 | - | + | + | - |
| 1I:2D:5 | - | + | - | - |
| 1I:2D:6 | - | + | - | - |
| 3D:2D:7 | - | + | - | - |
| 1I:2D:8 | - | + | - | - |
| 3D:9 | - | - | + | + |
| 3D:10 | - | - | + | + |
| 3D:11 | - | - | + | + |
| 3D:12 | - | - | + | + |
| 3D:13 | - | - | + | + |
| 1I:14 | - | + | + | - |
| 1I:15 | - | + | + | - |
| 1I:16 | - | + | + | - |
| 1I:17 | - | + | + | - |
| 1I:18 | - | + | + | - |
| 1I:19 | - | + | + | - |
| 1I:20 | - | + | + | - |
| 1I:21 | - | + | + | - |
| 1I:22 | - | + | + | - |
| 1I:23 | - | + | + | - |
| 1I:24 | - | + | + | - |
| 1I:25 | - | + | + | - |
| 1I:26 | - | + | + | - |
| 1I:27 | - | + | + | - |
| 3D:28 | - | - | + | + |
| 1I:29 | - | + | + | - |
| 1I:30 | - | + | + | - |
| 1I:31 | - | + | + | - |
| 1I:32 | - | + | + | - |
| 1I:14D:33 | - | + | + | - |
| 1I:14D:34 | - | + | + | - |
| 1I:14D:35 | - | + | + | - |
| 1I:36 | - | + | + | - |
| 2D:37 | - | + | + | - |
| 14D:38 | - | - | + | - |
| 1I:2D:39 | - | + | + | - |
| 1I:1I:40 | - | + | + | - |
| 1I:1I:41 | - | + | + | - |
| 1I:1I:42 | - | + | + | - |
| 1I:1I:43 | - | + | + | - |
| 1I:1I:44 | - | + | + | - |
| 1I:1I:45 | - | + | + | - |
| 1I:1I:46 | - | + | + | - |
| 3D:1I:47 | - | + | + | - |
| 3D:48 | - | + | + | + |
| 3D:2D:49 | - | + | + | - |
| WT:50 | - | - | - | + |
| WT:51 | - | - | - | + |
| WT:52 | - | - | - | + |
| WT:53 | - | - | - | + |
| WT:54 | - | - | - | + |
| WT:55 | - | - | - | + |
| WT:56 | - | - | - | + |
| WT:57 | - | - | - | + |
| WT:58 | - | - | - | + |
| WT:59 | - | - | - | + |

All reads with co-edits at sgRNA target 1+2 are out of frame. Nine of the mutant variants displayed a 3-nucleotide deletion that resulted in a single amino acid deletion without frame shift, the remaining 40 mutated/copies variants represent out of frame variants or truncated isoforms. D = deletion, I = Insertion, S = Substitution, WT = wild type.

**Supplementary Table 5: Summary of Next Generation Sequencing revealing the editing frequency at target site for sgRNA2 for 3 different allelic variants of the *MgCh* gene**

| Line | Types and percentages of editing events detected by next generation sequencing | | | | | | | | | | | | | | | | | | | | | | | |
| --- | --- | --- | --- | --- | --- | --- | --- | --- | --- | --- | --- | --- | --- | --- | --- | --- | --- | --- | --- | --- | --- | --- | --- | --- |
|  | V1 (5’- TGGCGCTGTGCAGATTGACC-3’) | | | | | | | | V2 (5’-TGG**T**GCTGTGCAGATTGACC-3’) | | | | | | | | V3 (5’-TGG**T**GCTGTGCAGA**C**TGACC-3’) | | | | | | | |
|  | Total | S | I | SI | D | SD | ID | SID | Total | S | I | SI | D | SD | ID | SID | Total | S | I | SI | D | SD | ID | SID |
| NG1 | 4.4 | 2.5 | 0.1 | 1.5 | 0.2 | 0.0 | 0.0 | 0.0 | 0.6 | 0.5 | 0.0 | 0.0 | 0.0 | 0.0 | 0.0 | 0.0 | 0.7 | 0.7 | 0.0 | 0.0 | 0.0 | 0.0 | 0.0 | 0.0 |
| NG2 | 6.3 | 2.4 | 0.2 | 3.1 | 0.5 | 0.0 | 0.0 | 0.0 | 0.6 | 0.6 | 0.0 | 0.0 | 0.0 | 0.0 | 0.0 | 0.0 | 0.8 | 0.7 | 0.0 | 0.0 | 0.0 | 0.0 | 0.0 | 0.0 |
| NG3 | 7.3 | 2.4 | 0.4 | 4.2 | 0.3 | 0.0 | 0.0 | 0.0 | 0.5 | 0.5 | 0.0 | 0.0 | 0.0 | 0.0 | 0.0 | 0.0 | 0.9 | 0.8 | 0.0 | 0.0 | 0.0 | 0.0 | 0.0 | 0.0 |
| HG1 | 28.0 | 1.5 | 0.1 | 25.9 | 0.4 | 0.0 | 0.0 | 0.0 | 0.6 | 0.5 | 0.0 | 0.0 | 0.0 | 0.0 | 0.0 | 0.0 | 0.6 | 0.5 | 0.0 | 0.0 | 0.0 | 0.0 | 0.0 | 0.0 |
| HG2 | 23.2 | 1.8 | 0.1 | 20.8 | 0.3 | 0.0 | 0.0 | 0.0 | 0.6 | 0.6 | 0.0 | 0.0 | 0.0 | 0.0 | 0.0 | 0.0 | 0.6 | 0.6 | 0.0 | 0.0 | 0.0 | 0.0 | 0.0 | 0.0 |
| HG3 | 2.7 | 2.3 | 0.1 | 0.3 | 0.1 | 0.0 | 0.0 | 0.0 | 0.7 | 0.7 | 0.0 | 0.0 | 0.0 | 0.0 | 0.0 | 0.0 | 0.9 | 0.8 | 0.1 | 0.0 | 0.0 | 0.0 | 0.0 | 0.0 |
| HY1 | 18.2 | 1.9 | 0.1 | 4.4 | 11.7 | 0.0 | 0.0 | 0.0 | 0.7 | 0.6 | 0.0 | 0.0 | 0.0 | 0.0 | 0.0 | 0.0 | 0.9 | 0.9 | 0.1 | 0.0 | 0.0 | 0.0 | 0.0 | 0.0 |
| HY2 | 17.2 | 1.8 | 0.1 | 2.7 | 12.4 | 0.0 | 0.0 | 0.0 | 0.7 | 0.6 | 0.0 | 0.0 | 0.0 | 0.0 | 0.0 | 0.0 | 0.8 | 0.8 | 0.1 | 0.0 | 0.0 | 0.0 | 0.0 | 0.0 |
| HY3 | 18.2 | 2.2 | 1.2 | 14.6 | 0.1 | 0.0 | 0.0 | 0.0 | 0.3 | 0.2 | 0.0 | 0.1 | 0.0 | 0.0 | 0.0 | 0.0 | 1.0 | 1.0 | 0.1 | 0.0 | 0.0 | 0.0 | 0.0 | 0.0 |
| Mean | 13.9 | 2.1 | 0.3 | 8.6 | 2.9 | 0.0 | 0.0 | 0.0 | 0.6 | 0.5 | 0.0 | 0.0 | 0.0 | 0.0 | 0.0 | 0.0 | 0.8 | 0.7 | 0.1 | 0.0 | 0.0 | 0.0 | 0.0 | 0.0 |
| WT | 3.2 | 2.8 | 0.2 | 0.1 | 0.1 | 0.0 | 0.0 | 0.0 | 0.5 | 0.5 | 0.0 | 0.0 | 0.0 | 0.0 | 0.0 | 0.0 | 0.7 | 0.6 | 0.0 | 0.0 | 0.0 | 0.0 | 0.0 | 0.0 |

Values represent edited reads in percent of total reads aligning with the target amplicon of *MgCh* at sgRNA1. S = substitution, I = Insertion, D = Deletion, SI = Substitution + Deletion, ID = Insertion + Deletion, SID = Substitution + Insertion + Deletion. SNPs from wildtype allelic variants in the sgRNA 2 target sequence shown in red, NG = Non-treated, green; HG = Heat treated, green; HY = heat treated, yellow; WT = wild type.

**Supplementary Table 6: Analysis of NGS reads that did not align to sgRNA target sequence due to long insertions or long deletions**

| Lines | sgRNA1 target site | | | | | sgRNA2 target site | | | |
| --- | --- | --- | --- | --- | --- | --- | --- | --- | --- |
|  | % Insertions | Average Length of Insertions | % Deletions | Average Length of Deletions | % Insertions | | Average Length of Insertions | % Deletions | Average Length of Deletions |
| NG1 | 0.08 ± 0.02 | 19.2 | 0.03 ± 0.03 | 1 | 0.02 ± 0.01 | | 9.3 | 0.74 ± 0.06 | 6.8 |
| NG2 | 2.36 ± 0.02 | 6.8 | 0.02 ± 0.0 | 4 | 0.01 ± 0.0 | | 3.9 | 1.58 ± 0.07 | 9.4 |
| NG3 | 0.13 ± 0.02 | 9.4 | 0.09 ± 0.03 | 1 | 0.02 ± 0.0 | | 1.8 | 1.9 ± 0.01 | 8.2 |
| HG1 | 6.51 ± 0.13 | 9.4 | 0.06 ± 0.0 | 9 | 0.03 ± 0.01 | | 3.4 | 0.67 ± 0.01 | 7.2 |
| HG2 | 4.68 ± 0.15 | 9.3 | 0.03 ± 0.0 | 8 | 0.02 ± 0.0 | | 2.4 | 0.71 ± 0.05 | 6.0 |
| HG3 | 0.05 ± 0.01 | 18.0 | 0.01 ± 0.01 | 1 | 0.01 ± 0.01 | | 1.0 | 0.51 ± 0.0 | 3.4 |
| HY1 | 0.09 ± 0.03 | 28.7 | 0.0 ± 0.0 | 1 | 0.01 ± 0.01 | | 2.1 | 18.83 ± 1.36 | 14.2 |
| HY2 | 0.07 ± 0.02 | 29.2 | 0.0 ± 0.0 | 0 | 0.03 ± 0.02 | | 1.8 | 19.39 ± 0.11 | 14.3 |
| HY3 | 0.05 ± 0.02 | 27.5 | 0.0 ± 0.0 | 9 | 0.09 ± 0.04 | | 3.6 | 15.34 ± 2.5 | 5.9 |
| MEAN | 1.6 | 17.5 | 0.026 | 3.7 | 0.026 | | 3.3 | 6.63 | 8.4 |
| WT | 0.05 ± 0.03 | 30.4 | 0.0 ± 0.0 | 0 | 0.02 ± 0.01 | | 1.5 | 0.37 ± 0.04 | 1.5 |
| LSD | 0.15 |  | 0.027 |  | 0.032 | |  | 1.49 | 2 |

Values represent NGS reads in which long deletions or long insertions prevent an alignment to the sgRNA target sequence in percent of total NGS reads for the specific target sequence. To record the reads with severely modified sgRNA targets while preventing alignment with the sgRNA sequence, all reads that contained a valid primer (C31 or C32) but did not contain the sgRNA sequence were examined. This included searching for an 11 bp conserved sequence that could be located downstream of the sgRNA (at positions 56 and 61 downstream of C31 or C32, respectively). This search was performed using a local alignment algorithm. % Insertions = percentage of reads with long insertions, % Deletions = percentage of reads with long deletions, LSD = Least Significant Difference. The value following the percentage is the standard deviation; p < 0.01
