# Supplementary material for: Multiallelic, Targeted Mutagenesis of Magnesium Chelatase With CRISPR/Cas9 Provides a Rapidly Scorable Phenotype in Highly Polyploid Sugarcane
Source: Front Genome Ed. 2021 Apr 29;3:654996. doi: 10.3389/fgeed.2021.654996 (PMC8525377; doi:10.3389/fgeed.2021.654996)
Supplement: Supplementary file 3 [file Data_Sheet_2.docx]

**Multiallelic, targeted mutagenesis of magnesium chelatase with CRISPR-Cas9 provides a rapidly scorable phenotype in highly polyploid sugarcane**

Ayman Eid^1,2^, Chakravarthi Mohan^2^, Sara Sanchez^1,2^, Duoduo Wang^1,2^, and Fredy Altpeter^1,2,3,4,*^

^1^Agronomy Department, IFAS, University of Florida, Gainesville, FL, USA. ^2^DOE Center for Advanced Bioenergy and Bioproducts Innovation, Gainesville, FL, USA. ^3^Genetics Institute, University of Florida, Gainesville, FL, USA. ^4^Plant Molecular and Cellular Biology Program, IFAS, Gainesville, FL, USA.

***Correspondence:** altpeter@ufl.edu

**Sequencing Key**

1. Sanger Sequencing:
2. Organization of the Sanger Data:

| **Mutant Line** | **Figure** | **Tables** |
| --- | --- | --- |
| HY1 | Figure 5 | Supplementary Table 2, Supplementary Table 3A & B, supplementary Table 4 |
| HY2, HY3, NG1, NG2, NG3, HG1, HG2, HG3 |  | Supplementary Figure 1 |

1. GenBank Accessions

| **ID** | **Accession** | **ID** | **Accession** | **ID** | **Accession** |
| --- | --- | --- | --- | --- | --- |
| NG1_1 | MW676179 | HY1_16 | MW676201 | HY1_40 | MW676189 |
| NG2_1 | MW676248 | HY1_17 | MW676202 | HY1_41 | MW676194 |
| NG2_2 | MW676250 | HY1_18 | MW676200 | HY1_42 | MW676191 |
| NG2_3 | MW676251 | HY1_19 | MW676206 | HY1_43 | MW676185 |
| NG3_1 | MW676247 | HY1_20 | MW676204 | HY1_44 | MW676188 |
| NG3_2 | MW676192 | HY1_21 | MW676199 | HY1_45 | MW676190 |
| HG1_1 | MW676183 | HY1_22 | MW676197 | HY1_46 | MW676193 |
| HG2_1 | MW676184 | HY1_23 | MW676207 | HY1_47 | MW676234 |
| HG3_1 | MW676186 | HY1_24 | MW676211 | HY1_48 | MW676237 |
| HY1_1 | MW676239 | HY1_25 | MW676210 | HY1_49 | MW676240 |
| HY1_2 | MW676233 | HY1_26 | MW676205 | HY1_50 | MW676209 |
| HY1_3 | MW676232 | HY1_27 | MW676235 | HY1_51 | MW676213 |
| HY1_4 | MW676226 | HY1_28 | MW676195 | HY1_52 | MW676214 |
| HY1_5 | MW676225 | HY1_29 | MW676196 | HY1_53 | MW676218 |
| HY1_6 | MW676231 | HY1_30 | MW676182 | HY1_54 | MW676222 |
| HY1_7 | MW676241 | HY1_31 | MW676198 | HY1_55 | MW676221 |
| HY1_8 | MW676224 | HY1_32 | MW676242 | HY1_56 | MW676220 |
| HY1_9 | MW676238 | HY1_33 | MW676244 | HY1_57 | MW676219 |
| HY1_10 | MW676228 | HY1_34 | MW676243 | HY1_58 | MW676216 |
| HY1_11 | MW676230 | HY1_35 | MW676208 | HY1_59 | MW676215 |
| HY1_12 | MW676229 | HY1_36 | MW676227 | HY2_1 | MW676246 |
| HY1_13 | MW676236 | HY1_37 | MW676245 | HY2_2 | MW676249 |
| HY1_14 | MW676203 | HY1_38 | MW676217 | HY3_1 | MW676187 |
| HY1_15 | MW676212 | HY1_39 | MW676223 | HY3_2 | MW676180 |
|  |  |  |  | HY3_3 | MW676181 |

1. Next generation sequencing
2. Code used in the analysis: <https://github.com/uf-icbr-bioinformatics/Sugarcane-MgCh>
3. NGS Data shown in:
   - Table 3
   - Supplementary Table 5
   - Supplementary Table 6
4. NCBI sequence read archive (SRA) access links

- NGS data are deposited under BioProject: PRJNA704370

<https://www.ncbi.nlm.nih.gov/sra/PRJNA704370>

- SRA link to fastq files

<https://trace.ncbi.nlm.nih.gov/Traces/sra/?study=SRP308269>

- NCBI link to the fastq files

<https://www.ncbi.nlm.nih.gov/sra?term=SRP308269>

| **ID** | **Accession** | **Sample Name** | **Link** |
| --- | --- | --- | --- |
| NG1A | SAMN18038754 | MgChI Mutant 1 Non-Heat treated Green Line Replicate A | [https://www.ncbi.nlm.nih.gov/sra/SRX10171704[accn]](https://www.ncbi.nlm.nih.gov/sra/SRX10171704%5baccn%5d) |
| NG1B | SAMN18038755 | MgChI Mutant 1 Non-Heat treated Green Line Replicate B | [https://www.ncbi.nlm.nih.gov/sra/SRX10171705[accn]](https://www.ncbi.nlm.nih.gov/sra/SRX10171705%5baccn%5d) |
| NG2A | SAMN18038756 | MgChI Mutant 2 Non-Heat treated Green Line Replicate A | [https://www.ncbi.nlm.nih.gov/sra/SRX10171716[accn]](https://www.ncbi.nlm.nih.gov/sra/SRX10171716%5baccn%5d) |
| NG2B | SAMN18038757 | MgChI Mutant 2 Non-Heat treated Green Line Replicate B | [https://www.ncbi.nlm.nih.gov/sra/SRX10171725[accn]](https://www.ncbi.nlm.nih.gov/sra/SRX10171725%5baccn%5d) |
| NG3A | SAMN18038758 | MgChI Mutant 3 Non-Heat treated Green Line Replicate A | [https://www.ncbi.nlm.nih.gov/sra/SRX10171726[accn]](https://www.ncbi.nlm.nih.gov/sra/SRX10171726%5baccn%5d) |
| NG3B | SAMN18038759 | MgChI Mutant 3 Non-Heat treated Green Line Replicate B | [https://www.ncbi.nlm.nih.gov/sra/SRX10171727[accn]](https://www.ncbi.nlm.nih.gov/sra/SRX10171727%5baccn%5d) |
| HG1A | SAMN18038760 | MgChI Mutant 1 Heat treated Green Line Replicate A | [https://www.ncbi.nlm.nih.gov/sra/SRX10171728[accn]](https://www.ncbi.nlm.nih.gov/sra/SRX10171728%5baccn%5d) |
| HG1B | SAMN18038761 | MgChI Mutant 1 Heat treated Green Line Replicate B | [https://www.ncbi.nlm.nih.gov/sra/SRX10171729[accn]](https://www.ncbi.nlm.nih.gov/sra/SRX10171729%5baccn%5d) |
| HG2A | SAMN18038762 | MgChI Mutant 2 Heat treated Green Line Replicate A | [https://www.ncbi.nlm.nih.gov/sra/SRX10171730[accn]](https://www.ncbi.nlm.nih.gov/sra/SRX10171730%5baccn%5d) |
| HG2B | SAMN18038763 | MgChI Mutant 2 Heat treated Green Line Replicate B | [https://www.ncbi.nlm.nih.gov/sra/SRX10171731[accn]](https://www.ncbi.nlm.nih.gov/sra/SRX10171731%5baccn%5d) |
| HG3A | SAMN18038764 | MgChI Mutant 3 Heat treated Green Line Replicate A | [https://www.ncbi.nlm.nih.gov/sra/SRX10171706[accn]](https://www.ncbi.nlm.nih.gov/sra/SRX10171706%5baccn%5d) |
| HG3B | SAMN18038765 | MgChI Mutant 3 Heat treated Green Line Replicate B | [https://www.ncbi.nlm.nih.gov/sra/SRX10171707[accn]](https://www.ncbi.nlm.nih.gov/sra/SRX10171707%5baccn%5d) |
| HY1A | SAMN18038766 | MgChI Mutant 1 Heat treated Yellow Line Replicate A | [https://www.ncbi.nlm.nih.gov/sra/SRX10171708[accn]](https://www.ncbi.nlm.nih.gov/sra/SRX10171708%5baccn%5d) |
| HY1B | SAMN18038767 | MgChI Mutant 1 Heat treated Yellow Line Replicate B | [https://www.ncbi.nlm.nih.gov/sra/SRX10171709[accn]](https://www.ncbi.nlm.nih.gov/sra/SRX10171709%5baccn%5d) |
| HY1C | SAMN18038768 | MgChI Mutant 1 Heat treated Yellow Line Replicate C | [https://www.ncbi.nlm.nih.gov/sra/SRX10171710[accn]](https://www.ncbi.nlm.nih.gov/sra/SRX10171710%5baccn%5d) |
| HY1D | SAMN18038769 | MgChI Mutant 1 Heat treated Yellow Line Replicate D | [https://www.ncbi.nlm.nih.gov/sra/SRX10171711[accn]](https://www.ncbi.nlm.nih.gov/sra/SRX10171711%5baccn%5d) |
| HY1E | SAMN18038770 | MgChI Mutant 1 Heat treated Yellow Line Replicate E | [https://www.ncbi.nlm.nih.gov/sra/SRX10171712[accn]](https://www.ncbi.nlm.nih.gov/sra/SRX10171712%5baccn%5d) |
| HY2A | SAMN18038771 | MgChI Mutant 2 Heat treated Yellow Line Replicate A | [https://www.ncbi.nlm.nih.gov/sra/SRX10171713[accn]](https://www.ncbi.nlm.nih.gov/sra/SRX10171713%5baccn%5d) |
| HY2B | SAMN18038772 | MgChI Mutant 2 Heat treated Yellow Line Replicate B | [https://www.ncbi.nlm.nih.gov/sra/SRX10171714[accn]](https://www.ncbi.nlm.nih.gov/sra/SRX10171714%5baccn%5d) |
| HY2C | SAMN18038773 | MgChI Mutant 2 Heat treated Yellow Line Replicate C | [https://www.ncbi.nlm.nih.gov/sra/SRX10171715[accn]](https://www.ncbi.nlm.nih.gov/sra/SRX10171715%5baccn%5d) |
| HY2D | SAMN18038774 | MgChI Mutant 2 Heat treated Yellow Line Replicate D | [https://www.ncbi.nlm.nih.gov/sra/SRX10171717[accn]](https://www.ncbi.nlm.nih.gov/sra/SRX10171717%5baccn%5d) |
| HY2E | SAMN18038775 | MgChI Mutant 2 Heat treated Yellow Line Replicate E | [https://www.ncbi.nlm.nih.gov/sra/SRX10171721[accn]](https://www.ncbi.nlm.nih.gov/sra/SRX10171721%5baccn%5d) |
| HY3C | SAMN18038776 | MgChI Mutant 3 Heat treated Yellow Line Replicate A | [https://www.ncbi.nlm.nih.gov/sra/SRX10171719[accn]](https://www.ncbi.nlm.nih.gov/sra/SRX10171719%5baccn%5d) |
| HY3D | SAMN18038777 | MgChI Mutant 3 Heat treated Yellow Line Replicate B | [https://www.ncbi.nlm.nih.gov/sra/SRX10171720[accn]](https://www.ncbi.nlm.nih.gov/sra/SRX10171720%5baccn%5d) |
| HY3E | SAMN18038778 | MgChI Mutant 3 Heat treated Yellow Line Replicate C | [https://www.ncbi.nlm.nih.gov/sra/SRX10171718[accn]](https://www.ncbi.nlm.nih.gov/sra/SRX10171718%5baccn%5d) |
| WTA | SAMN18038779 | Wild-Type Replicate A | [https://www.ncbi.nlm.nih.gov/sra/SRX10171722[accn]](https://www.ncbi.nlm.nih.gov/sra/SRX10171722%5baccn%5d) |
| WTB | SAMN18038780 | Wild-Type Replicate B | [https://www.ncbi.nlm.nih.gov/sra/SRX10171723[accn]](https://www.ncbi.nlm.nih.gov/sra/SRX10171723%5baccn%5d) |
| WTC | SAMN18038781 | Wild-Type Replicate C | [https://www.ncbi.nlm.nih.gov/sra/SRX10171724[accn]](https://www.ncbi.nlm.nih.gov/sra/SRX10171724%5baccn%5d) |
